# REEPs Are Membrane Shaping Adapter Proteins That Modulate Specific G Protein-Coupled Receptor Trafficking by Affecting ER Cargo Capacity

**DOI:** 10.1371/journal.pone.0076366

**Published:** 2013-10-02

**Authors:** Susann Björk, Carl M. Hurt, Vincent K. Ho, Timothy Angelotti

**Affiliations:** 1 Department of Pharmacology, Drug Development and Therapeutics, Institute of Biomedicine, University of Turku, Turku, Finland; 2 Department of Anesthesia/CCM, Stanford University Medical School, Stanford, California, United States of America; German Institute of Human Nutrition Potsdam-Rehbruecke, Germany

## Abstract

Receptor expression enhancing proteins (REEPs) were identified by their ability to enhance cell surface expression of a subset of G protein-coupled receptors (GPCRs), specifically GPCRs that have proven difficult to express in heterologous cell systems. Further analysis revealed that they belong to the Yip (Ypt-interacting protein) family and that some REEP subtypes affect ER structure. Yip family comparisons have established other potential roles for REEPs, including regulation of ER-Golgi transport and processing/neuronal localization of cargo proteins. However, these other potential REEP functions and the mechanism by which they selectively enhance GPCR cell surface expression have not been clarified. By utilizing several REEP family members (REEP1, REEP2, and REEP6) and model GPCRs (α2A and α2C adrenergic receptors), we examined REEP regulation of GPCR plasma membrane expression, intracellular processing, and trafficking. Using a combination of immunolocalization and biochemical methods, we demonstrated that this REEP subset is localized primarily to ER, but not plasma membranes. Single cell analysis demonstrated that these REEPs do not specifically enhance surface expression of all GPCRs, but affect ER cargo capacity of specific GPCRs and thus their surface expression. REEP co-expression with α2 adrenergic receptors (ARs) revealed that this REEP subset interacts with and alter glycosidic processing of α2C, but not α2A ARs, demonstrating selective interaction with cargo proteins. Specifically, these REEPs enhanced expression of and interacted with minimally/non-glycosylated forms of α2C ARs. Most importantly, expression of a mutant REEP1 allele (hereditary spastic paraplegia SPG31) lacking the carboxyl terminus led to loss of this interaction. Thus specific REEP isoforms have additional intracellular functions besides altering ER structure, such as enhancing ER cargo capacity, regulating ER-Golgi processing, and interacting with select cargo proteins. Therefore, some REEPs can be further described as ER membrane shaping adapter proteins.

## Introduction

In an attempt to find proteins that would enhance heterologous (e.g. HEK293) cell surface expression of olfactory receptors (OR), Matsunami and colleagues identified a new family of six proteins that they termed “receptor expression-enhancing proteins” or REEPs [1]. They demonstrated that co-expression of REEP1 led to enhanced functional surface expression for some, but not all ORs or G-protein coupled receptors (GPCRs). Similarly, REEPs have been shown to enhance heterologous expression of taste receptors (TR) [2,3], leading to the hypothesis that REEPs enhanced expression of a variety of poorly expressed GPCRs, possibly as chaperones or co-receptors. The mechanism by which REEPs selectively enhance expression of only a subset of GPCRs has not been determined. In addition, REEP1 mutations were found to be a genetic cause for the neurodegenerative disorder hereditary spastic paraplegia (HSP) [4,5]. Over fifty percent of North American HSP cases are due to mutations in M1-spastin, atlastin-1, or REEP1, proteins that are important determinants of curved endoplasmic reticulum (ER) tubule formation, elongation, and microtubule network interactions (reviewed in reference [6]).

A sequence comparison revealed that REEPs are homologous to yeast (Yop1) and barley (HVA22) proteins, thus reclassifying them as Yip (Ypt interacting protein) family members. They have been alternatively named the Yip2 family [7]. Yip family members, including Yop1 and HVA22, have been shown to interact directly with Rab GTPases, SNAREs, and ER/Golgi vesicle proteins to regulate intracellular trafficking and targeting of cargo proteins within yeast and neurons [8-15]. REEP1, REEP2, REEP5 (DP1), and Yop1 have been shown to affect ER structure [16-19], but despite their characterization as ER shaping proteins, less is known about how they regulate GPCR or other cargo transport and membrane expression [1,2].

To further investigate and clarify the roles and mechanisms of REEP modulation of cargo protein trafficking, we utilized α2A and α2C adrenergic receptors (ARs) as model GPCRs. Despite being highly homologous, α2A and α2C ARs have different neuronal localization and expression patterns [20-22]. For example, heterologous expression of α2C ARs in non-neuronal cells is more difficult to achieve than with α2A ARs. To further elucidate REEP effects, we applied a variety of immunofluorescent, biochemical, and quantitative FACS methods, previously developed for analysis of GPCR trafficking motifs, to our analysis of REEP function [23]. By utilizing these methods, we have been able to gain insight into REEP/GPCR interactions and build upon previous observations by others [1-3].

By examining co-expression of wild-type and HSP mutant REEPs with α2A and α2C ARs, we demonstrated that co-expression of a subset of REEPs enhances ER cargo capacity, in order to selectively modulate membrane expression of some GPCRs. Second, these REEP isoforms are ER resident proteins that can interact selectively with specific GPCRs; they can differentiate between cargo proteins. Third, specific REEP co-expression can affect ER to Golgi trafficking of α2C ARs, enhancing the expression of a minimally glycosylated form. Lastly, this α2C AR form interacts with these REEPs and a HSP REEP1 mutation lacking the carboxyl terminus shows no interaction. This study lends support to the hypothesis that some REEP isoforms, like other Yip family members, regulate intracellular trafficking by affecting ER membrane structure, cargo capacity, and by acting as adapter proteins. Thus, they meet the criteria for being reclassified as ER membrane shaping adapter proteins [24].

## Results

REEP family members were originally discovered and further described in terms of their ability to enhance plasma membrane or functional expression of GPCRs, namely ORs and bitter or sweet TRs [1-3]. However, contrasting discoveries were made about REEPs, such as their intracellular localization (e.g. mitochondria vs. ER) [4,16,18], and their mechanisms of actions were not completely elucidated. Also, two subfamilies of REEP proteins (REEP1-4 and REEP5-6/Yop1) have been described based upon structural and sequence homology [16] and detailed analyses of REEP5-6/Yop1 effects on GPCR expression have not been described.

To further clarify these issues and also investigate possible Yip-like affects on cargo protein trafficking, we examined the interactions between a subset of REEPs and model GPCRs, α2A and α2C ARs. These GPCRs are highly homologous, however α2C ARs show some similarity to ORs in that they display ER retention when expressed in HEK293 cells; α2A ARs are efficiently expressed on the cell surface [20,25]. Also, α2A and α2C ARs traffic to different locations within cultured sympathetic ganglion neurons (SGN), pre-synaptic and extra-synaptic respectively, suggesting differences in intracellular trafficking characteristics [21]. We chose a subset of REEP proteins for study (REEP1, REEP2, and REEP6) because prior research demonstrated that members of this subset can alter ER structure [16,17]. Also, this subset was chosen because REEP1 has been studied most extensively and is linked to HSP [16], REEP2 has shown differential effects on functional expression of various GPCRs [3], and REEP6 is a member of the least studied subfamily REEP5-6/Yop1. To enable direct comparisons to prior research and to allow for FACS-based analyses, we utilized a carboxyl terminus epitope-tagged REEP (Flag) and amino terminus epitope-tagged α2 AR (HA) constructs for all of our studies.

### REEPs are ER resident proteins

By studying Flag and HA-tagged REEP constructs, it was initially suggested that REEP1 and REEP2 could traffic to the plasma membrane [1,3], though later immunocytochemical and immunoblotting methods using specific REEP1 antisera suggested that REEP1 was localized to mitochondrial membranes [4]. Furthermore, an examination of REEP1 expression in COS7 cells and cortical neurons demonstrated co-localization with ER resident proteins and microtubules [16], thus leading to confusion. To validate our antibody-based methodologies, we first examined subcellular expression of α2 ARs and REEP1, REEP2, and REEP6 (REEP1/2/6) in transfected HEK293A cells. HEK293A cells were chosen because they are flatter and have a smaller nuclear to cytoplasmic ratio than wild-type HEK293 cells, thus improving immunofluorescent analysis.

**Figure 1:**
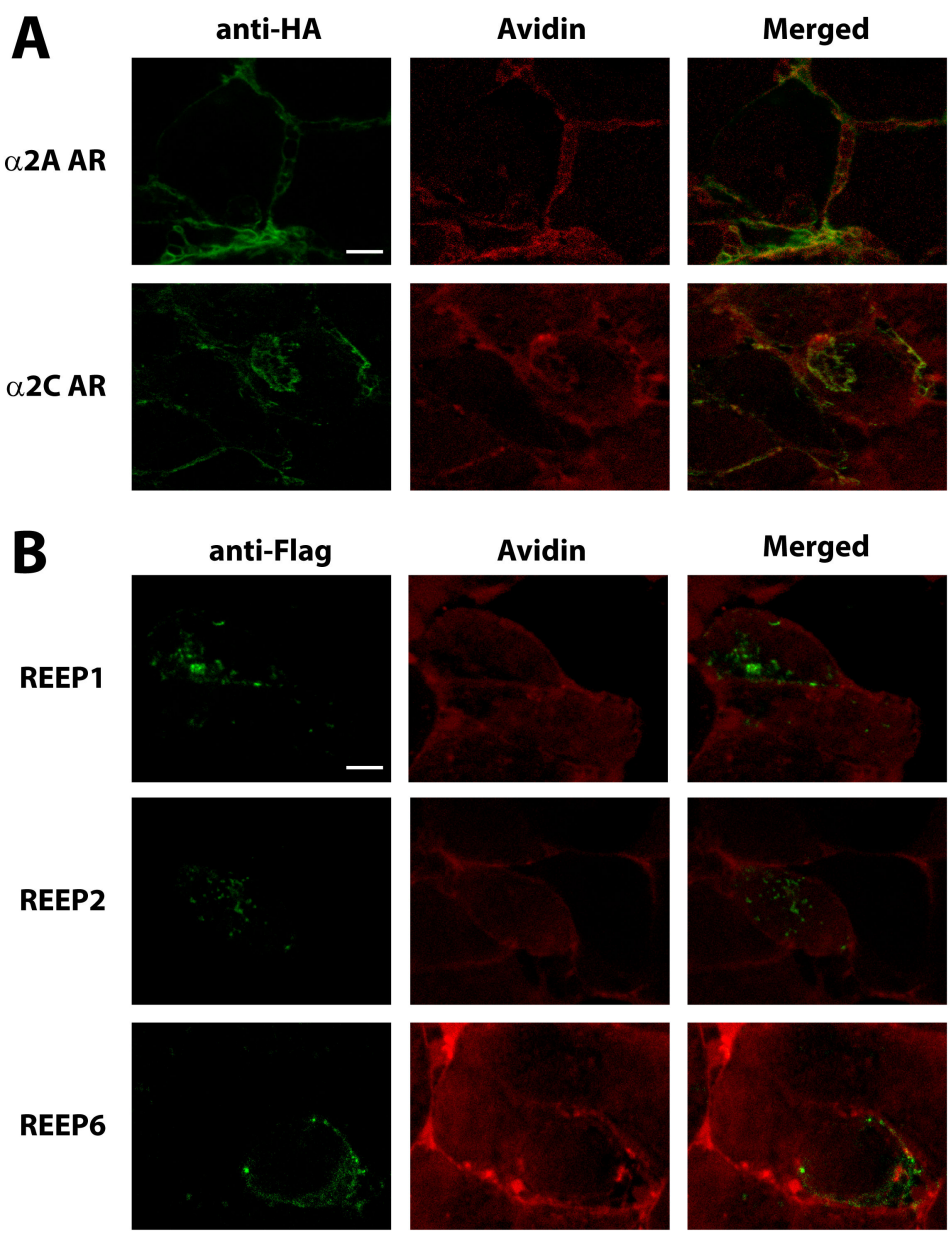
Confocal plasma membrane localization of α2 ARs and REEPs. HEK293A cells were transfected individually with either HA-α2A AR, HA-α2C AR, Flag-REEP1, Flag-REEP2, or Flag–REEP6 cDNA. Forty-eight hrs post-transfections, cells were biotinylated *in* *vivo* to label plasma membrane proteins. Subsequently, cells were fixed with 4% PFA, but not permeabilized, labeled with various immunofluorescent conjugates, and examined by confocal microscopy. HA- α2 ARs were labeled with 16B12-Alexa 488 (anti-HA) and Flag-REEPs were identified by M2-FITC (anti-Flag) antisera respectively (Left). Plasma membranes were identified by Alexa 594-conjugated avidin (Middle). Merged images are shown (Right). **A**. α2 AR immunolabeling of non-permeabilized cells demonstrated plasma membrane co-localization of α2A and α2C ARs, as evidenced by co-localization with Alexa 594-conjugated avidin. **B**. No REEP co-localization was seen with Alexa 594-conjugated avidin, suggesting that REEPs were not localized to the plasma membrane. REEPs were localized intracellularly, within the avidin delineated plasma membrane. REEP immunolabeling of non-permeabilized cells did reveal intracellular staining, despite the absence of permeabilization, due to the ability of antibodies to gain intracellular entry following PFA fixation [27]. Note the absence of immunolabeling of intracellularly localized α2A or α2C ARs, due to the lack of cell permeabilization (as seen in Figure 5). A more complete description of this methodology and findings can be found in **Results**. Representative of three separate transfections. Scale bars: 25 µm.

To more clearly delineate plasma membranes, we performed live cell biotinylation of transfected cells followed by confocal microscopy with fluorescent-conjugated avidin and anti-Flag and anti-HA antibodies in non-permeabilized cells (Figure 1). We examined non-permeabilized cells to enhance membrane delineation of α2 ARs and allow for clearer co-localization with avidin. Both α2A and α2C ARs can be seen at the plasma membrane, co-localized with avidin. Immunofluorescent analysis of transfected α2A and α2C ARs in non-permeabilized HEK293A cells revealed plasma membrane expression, similar to that seen previously. The intensity of α2C AR fluorescence at the plasma membrane was less than seen with α2A ARs, consistent with lower plasma membrane expression seen after heterologous expression in HEK293A cells [22,25,26]. Note the absence of intracellular staining of α2 ARs in non-permeabilized cells (as compared to intracellular α2 AR staining seen in permeabilized cells below). However, plasma membrane localization was not seen with REEP1/2/6. With the confocal images focused on the plasma membrane, all three REEPs studied appeared to be limited to the cytoplasmic compartment, contained within the borders of the plasma membrane. Intracellular staining of REEP1/2/6 was visible despite the lack of permeabilization, since antibodies can gain limited intracellular entry into PFA fixed, but non-permeabilized cells and thus label cytoplasmic epitopes (e.g. carboxyl terminus Flag epitope-tagged REEPs) (discussed further below) [27].

**Figure 2:**
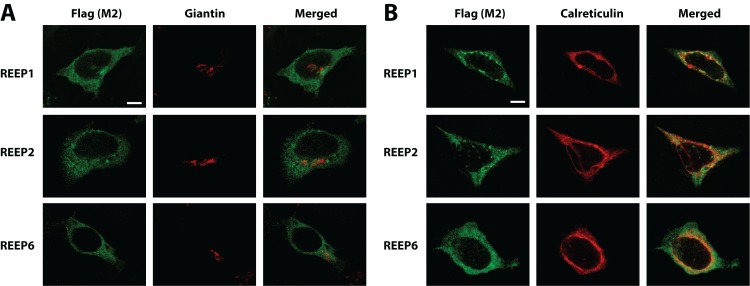
Confocal intracellular immunolocalization of REEPs. HEK293A cells were transfected with Flag-REEP1, -REEP2, or -REEP6. Forty-eight hrs post-transfection, cells were fixed with 4% PFA, permeabilized, and examined by confocal microscopy. REEPs were stained with M2 antibody (anti-Flag). Golgi and ER compartments were stained for giantin and calreticulin expression respectively. **A**: REEP1/2/6 (Left) did not co-localize with the Golgi compartment, delineated by the Golgi marker giantin (Middle), as seen in merged images (Right). **B**: REEP1/2/6 staining (Left) delineated an intracellular reticular pattern that overlapped with the ER marker calreticulin (Middle), as seen in merged images (Right). All REEPs studied identified a reticular pattern more diffuse than that labeled by anti-calreticulin antisera, suggesting a possible alteration in calreticulin distribution upon REEP co-expression (Right). Representative of three separate transfections. Scale bars: 25 µm.

Next, we used confocal microscopy to examine the subcellular localization of REEP1/2/6 in permeabilized HEK293A cells, compared with ER and Golgi marker proteins (Figure 2). Confocal images were focused on either the ER or Golgi planes, to enhance co-localization. As had been seen previously for REEP1, all three REEPs localized primarily to an intracellular compartment, co-localizing with calreticulin (ER) but not giantin (Golgi), suggesting that REEP1/2/6 are ER resident proteins. However, there was not extensive overlap between these REEPs and calreticulin, and the regions of highest REEP expression did not co-localize with calreticulin. The ER is a complex structure with multiple subdomains and functions [28] and it has been demonstrated that known ER resident proteins do not always completely co-localize within various ER subdomains, possibly accounting for the lack of co-localization between REEPs and calreticulin in some ER domains [29].

**Figure 3:**
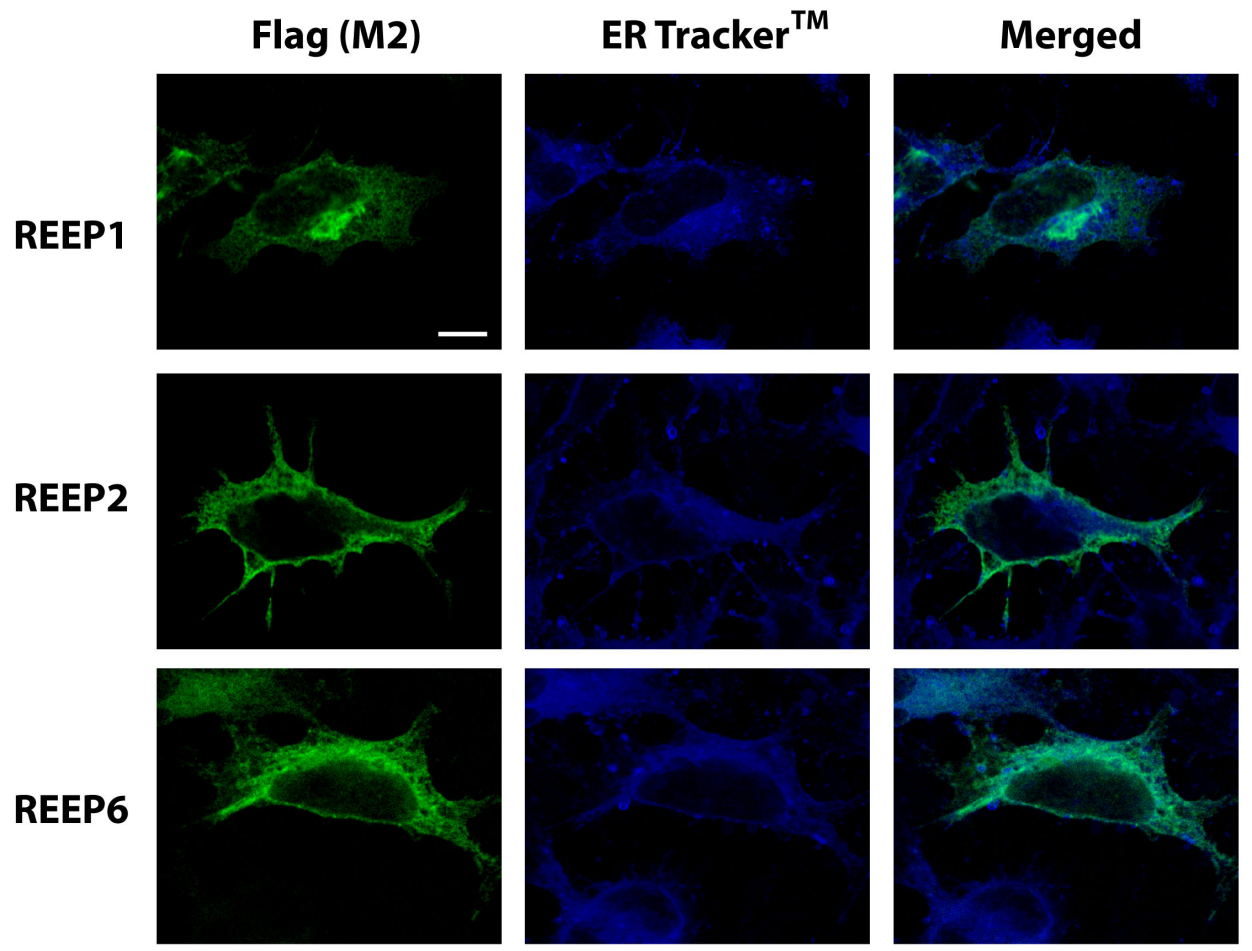
Confocal ER localization of REEPs with ER Tracker™ dye. HEK293A cells were transfected with Flag-REEP1, -REEP2, or -REEP6. Forty-eight hrs post-transfection, cells were fixed with 4% PFA, permeabilized, and examined by confocal microscopy. The ER was identified by staining with the ER-specific dye ER Tracker™ Blue/White DPX, which is retained within the ER lumen, thus labeling the ER tubular network (29). REEPs were stained with M2-Alexa 488 antibody (anti-Flag). REEP1/2/6 staining (Left) delineated an intracellular reticular pattern that showed extensive overlap with the ER luminal network (Middle), as seen in merged images (Right). Areas of punctate REEP expression likely represent areas of focal accumulation within the ER and confocal cross-sections of ER tubules. REEP1 demonstrated focal accumulation near the nucleus (arrow), whereas REEP2 was not found in all ER Tracker™-labeled ER regions (arrow), suggesting the existence of possible REEP/ER subdomains. Representative of three separate transfections. Scale bars: 25 µm.

Since REEPs are known to alter ER structure and possibly ER subdomains [16,17], we next used an ER-specific dye to localize the ER structure and look for REEP co-localization (Figure 3). ER Tracker™ Blue/White DPX is a dye that is specifically retained within the ER lumen, thus it is not dependent upon any ER membrane resident proteins for localization [30]. Examination of REEP1/2/6 in transfected cells stained with ER Tracker™ revealed extensive co-localization throughout the ER tubular network, further supporting an ER localization for REEP1/2/6. Since the ER tubular network is a cage-like structure [31], punctate regions of REEP expression can be seen, representing a confocal slice through an ER tubule. Inspection of REEP1 expression demonstrated focal accumulation near the nucleus, whereas REEP2 was not co-localized to all regions labeled by ER Tracker™, consistent with possible differential REEP expression within ER subdomains.

**Figure 4:**
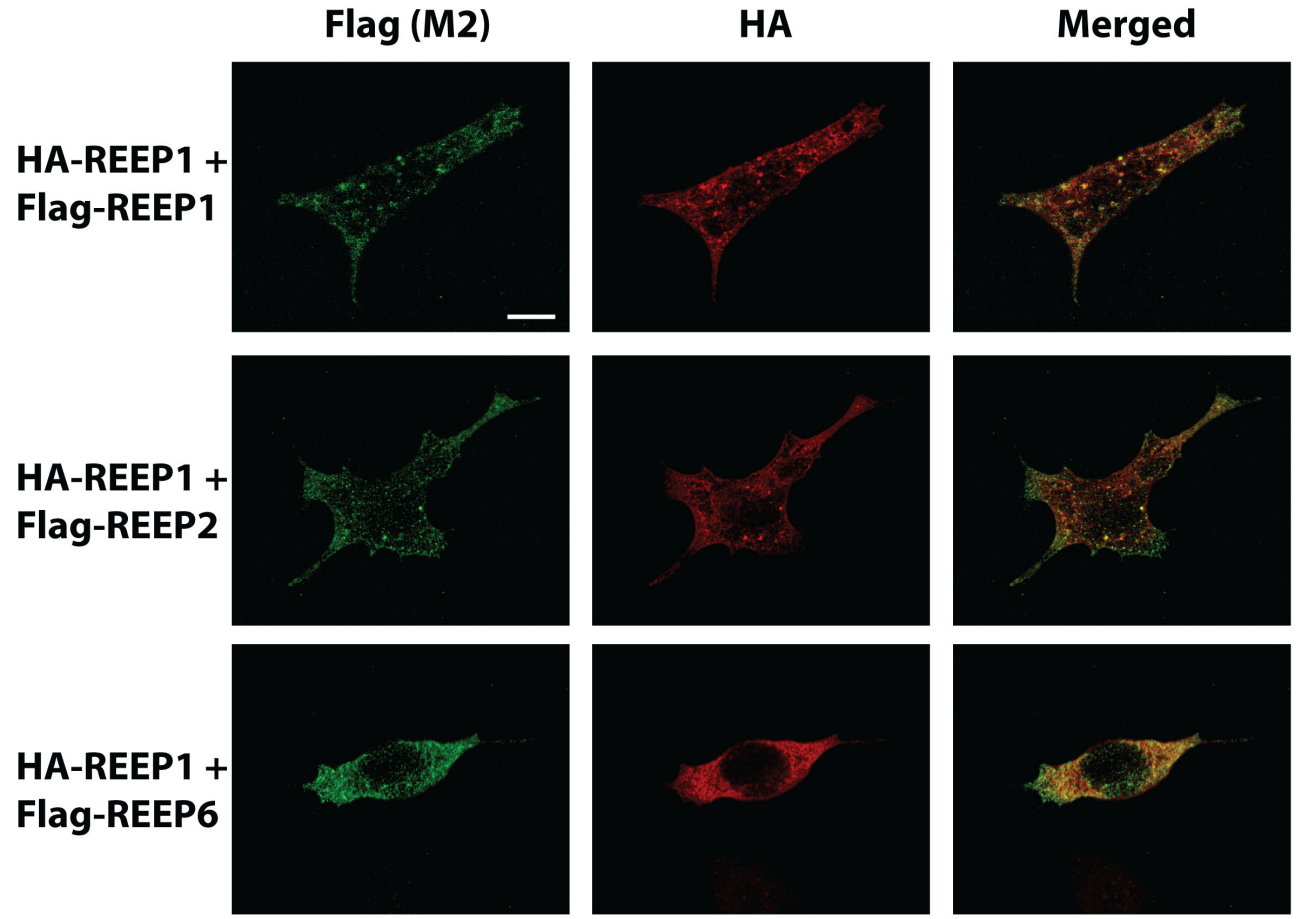
Confocal REEP isoform co-localization. HEK293A cells were transfected with HA-REEP1 (carboxyl terminus HA-epitope tag) and either Flag-REEP1, Flag-REEP2, or Flag–REEP6 (carboxyl terminus Flag-epitope tag) cDNA. Forty-eight hrs post-transfections, cells were fixed with 4% PFA, permeabilized, and examined by confocal microscopy. Flag-REEPs (Left) were identified by FITC-M2 (anti-Flag) antisera and HA-REEP1 (Middle) was labeled with Alexa 594 anti-HA antisera. Merged images are shown (Right). Co-expression of HA-REEP1 and Flag-REEP1 showed tremendous overlap, as expected. Flag-REEP2 and HA-REEP1 showed multiple punctate regions of co-expression. However, Flag-REEP2 also exhibited further extensions devoid of HA-REEP1 expression. Lastly, Flag-REEP6 and HA-REEP1 demonstrated a large degree of co-localization. Representative of three separate transfections. Scale bars: 25 µm.

To quantify the relationship between REEP expression within the ER, Pearson correlation coefficients for REEP1/2/6 and ER Tracker™ confocal immunofluorescent images were calculated (Table 1). The Global Pearson’s Correlation values were > 0.60, which suggested a significant correlation between REEP1/2/6 and ER Tracker™ co-localization [32]. Subsequently co-localization coefficients m_1_ (extent of ER Tracker™ co-localized with REEPs) and m_2_ (extent of REEP co-localized with ER Tracker™) were calculated. Calculated m_1_ values demonstrated that not all ER Tracker™ co-localized with REEP1/2/6 (90.4, 73.2, and 91.9% respectively), demonstrating that REEPs did localize to the majority of the ER structure with some subdomains of the ER lacking REEP expression, as suggested by the confocal images. Conversely, calculated m_2_ values demonstrated that REEP1/2/6 almost completely co-localized to the ER (98.3, 98.8, and 94.3% respectively), verifying that REEP1/2/6 were ER resident proteins. Since REEP1/2/6 displayed limited co-localization with calreticulin, but were well co-localized to the ER tubular network (as delineated by ER Tracker™ Blue/White DPX), we investigated whether REEP1, REEP2, and REEP6 localized to similar ER subdomains (Figure 4). A carboxyl terminus HA-epitope tagged REEP1 construct was transfected into HEK293A cells, along with Flag-tagged-REEP1, -REEP2, and –REEP6, and examined by confocal microscopy. HA-REEP1 and Flag-REEP1 revealed co-localization within the ER, as would be expected, suggesting that the different epitopes did not affect intracellular localization of REEP1. Additionally, Flag-REEP2 and –REEP6 also co-localized extensively with HA-REEP1, suggesting that REEP1/2/6 do not localize to different ER subdomains.

**Table 1:** Pearson’s correlation coefficients for REEP co-localization with ER Tracker™.

	**REEP1**	**REEP2**	**REEP6**
	**& ER Tracker™**	**& ER Tracker™**	**& ER Tracker™**
**Global Pearson’s**			
**Correlation:**	0.649	0.669	0.732
**Co-localization**			
**Coefficient (m_1_):**	0.904	0.732	0.919
**Co-localization**			
**Coefficient (m_2_):**	0.983	0.988	0.943

m_1_: Represents the extent of ER Blue/White Tracker co-localization with REEP protein. m_2_: Represents the extent of REEP protein co-localization with ER Blue/White Tracker.

Pearson’s correlation coefficients and co-localization coefficients (m_1_ and m_2_) were calculated from REEP and ER Blue/White Tracker™ dye confocal images (Figure 3), as described in Materials and Methods.

**Figure 5:**
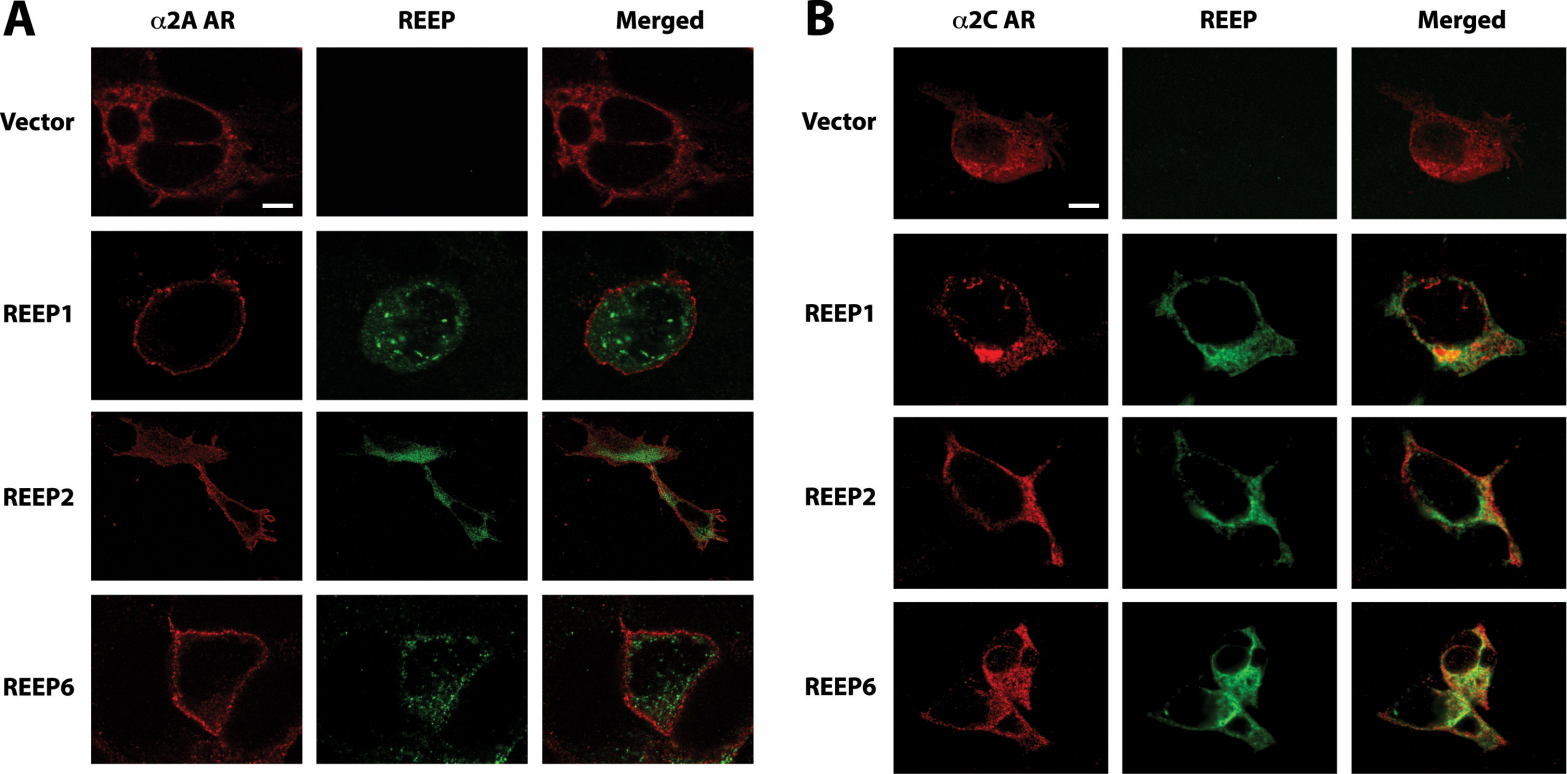
Confocal co-localization of α2 ARs and REEPs in permeabilized cells. HEK293A cells were co-transfected with HA-α2A or –α2C AR cDNAs and either empty vector (pcDNA3.1), Flag-REEP1, -REEP2, or –REEP6 cDNAs. Cells were fixed with 4% PFA, permeabilized, and examined by confocal microscopy forty-eight hrs post-transfection. α2A and α2C ARs were stained with anti-HA mAb (16B12) and Alexa 594 conjugated-anti mouse secondary antisera; REEPs were stained with rabbit anti-Flag polyclonal antisera and Alexa 488 conjugated anti-rabbit secondary antisera. Confocal images were focused to include ER membrane planes and allow imaging of REEP and α2 AR expression. **A**. α2A ARs demonstrated predominant plasma membrane expression as described previously [25] (Left). Immunolabeling of REEPs (Middle) identified an intracellular reticular/punctate pattern that did not overlap with plasma membrane localized α2A ARs (Right). **B**. Immunolabeling for α2C ARs (Left) demonstrated a large intracellular pool of receptor, as described previously [25]. REEPs (Middle) were localized to the intracellular space (ER) and small amount of overlap with α2C ARs was detected (Right). Punctate areas of overlap between α2C ARs and REEPs can be seen, which were not as evident with α2A ARs. Absence of either α2 AR (vector control) did not alter REEP localization. Representative of three separate transfections. Scale bars: 25 µm.

Previously, it had been suggested that REEPs may act as co-receptors for ORs, leading to REEP expression at the cell membrane [1]. To investigate if either α2 AR subtype possibly interacted with REEPs, we examined co-localization of α2A and α2C ARs with REEP1/2/6 by confocal microscopy of transfected HEK293A cells (Figure 5). To enhance our ability to detect co-localization and possible interactions, we focused on the cell plane that allowed for simultaneous detection of both REEPs and α2 ARs (ER membrane). All REEPs examined did not demonstrate apparent co-localization with α2A AR within the ER membrane. However, α2C ARs and REEP1/2/6 did show some small areas of co-localization and thus possible sites of interaction within the ER, which is the predominant site of α2C AR localization [25]. Since α2A ARs are predominantly expressed at the plasma membrane [25], whereas REEP1/2/6 are ER resident proteins, more clear confocal analysis of the plasma membrane was performed for α2A ARs (Figure S1). Again, no co-localization of α2A ARs and REEP1/2/6 was observed. Minimal amounts of REEP1/2/6 were visualized within the borders of the plasma membrane, as defined by α2A ARs (described previously in Figure 1).

**Figure 6:**
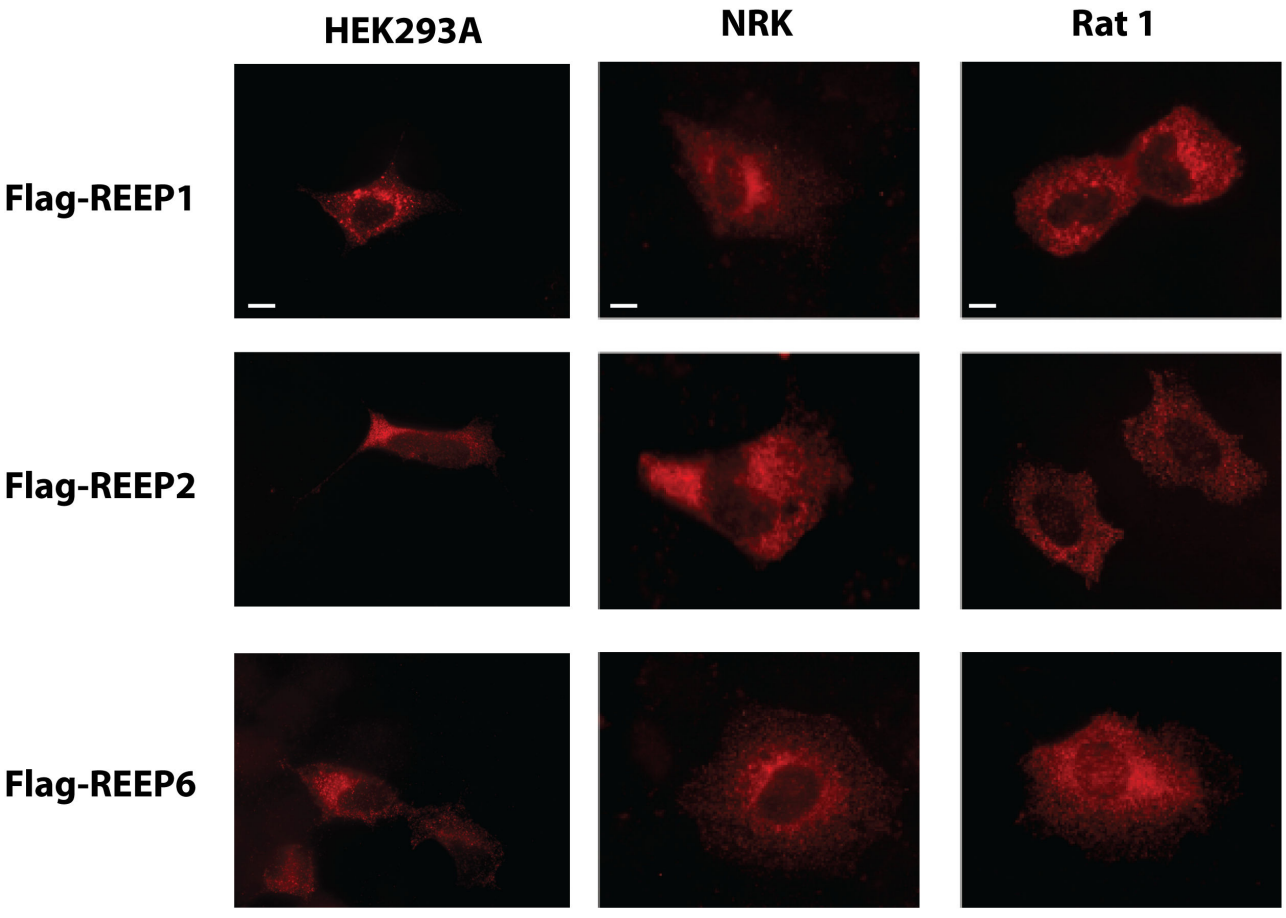
REEP expression in non-permeabilized HEK293A, NRK, and Rat1 cells. HEK293A, NRK, and Rat1 cells were transfected with Flag-REEP1, -REEP2, or –REEP6. Forty-eight hrs later, cells were fixed with 4% PFA, but not permeabilized, for immunofluorescent labeling. Flag-REEPs were labeled with M2 (anti-Flag) antisera and identified with Alexa 594-conjugated goat anti-mouse secondary antibody. Note the similar strong peri-nuclear and intracellular reticular staining pattern seen in all three cell lines. Representative of three separate transfections. Scale bars: 10 µm.

HEK293A cells have been genetically modified to enhance the production of transfected cDNA-encoded proteins, which may alter localization. However, transfection of REEP1/2/6 into Rat1 and NRK cells, which have not been modified to enhance protein production, showed similar intracellular staining patterns as HEK293A cells (Figure 6). In all three cell types, a fine trabecular network originating from a perinuclear shadow can be seen, consistent with an intracellular compartment localization. This staining pattern is similar to that seen when REEP1/2/6 expression was examined with the ER Tracker™ Blue/White DPX dye (Figure 3). Therefore, localization of heterologously expressed REEPs did not appear to depend upon the cell-type studied or protein expression levels.

### Biochemical analysis of REEP membrane localization

**Figure 7:**
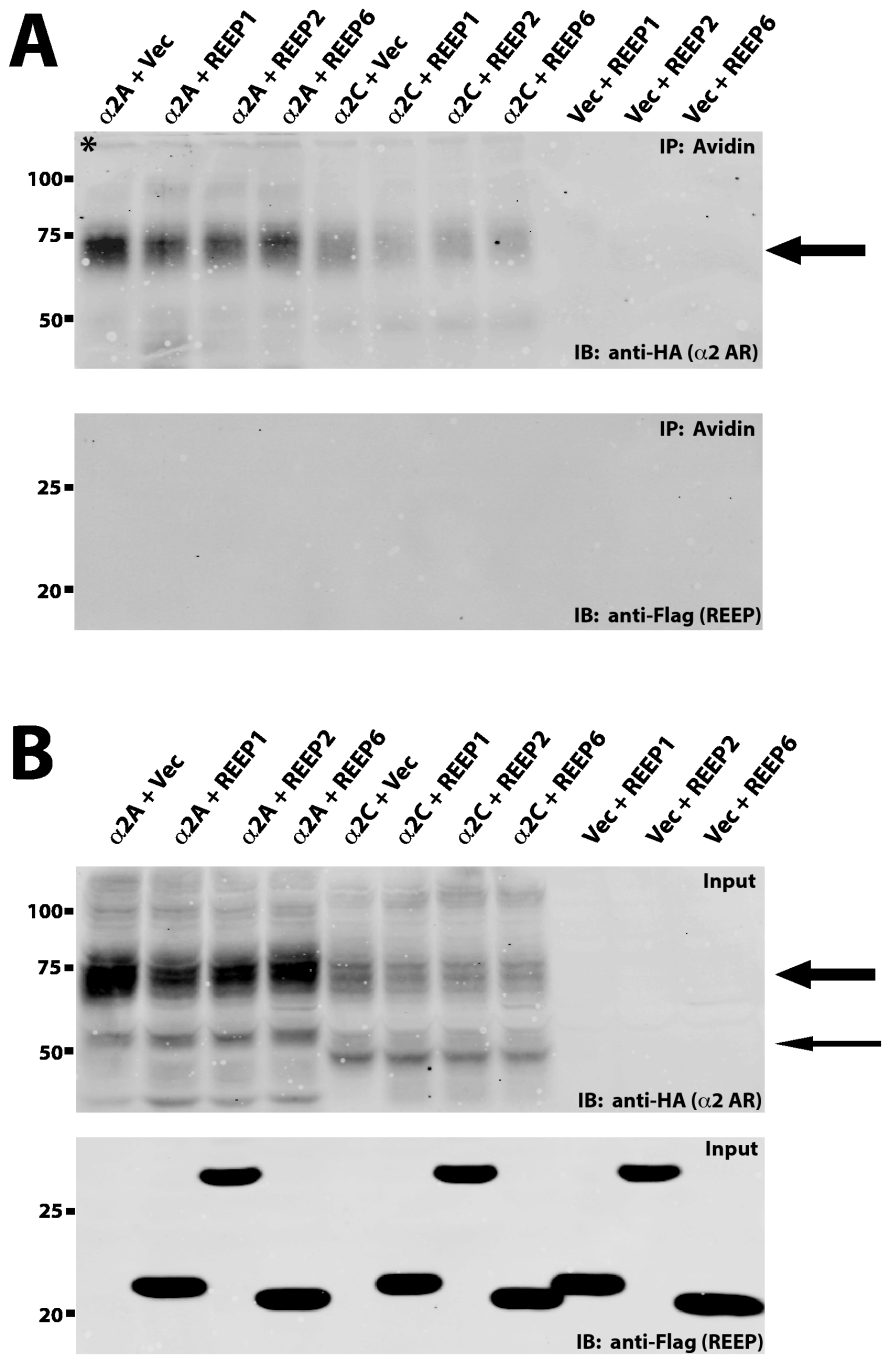
In vivo biotinylation analysis of REEP plasma membrane expression. To determine if REEPs were expressed at the plasma membrane, HEK293A cells were transfected with Flag-REEP1, Flag-REEP2, or Flag–REEP6 cDNA with or without co-transfected HA-α2A AR or HA-α2C AR cDNAs. Forty-eight hrs post-transfections, cells were treated with the biotinylating reagent EZ-Link Sulfo-NHS-SS-Biotin (Pierce), total cell lysates were isolated, and biotinylated proteins were precipitated by incubation with avidin-agarose. Avidin precipitated proteins and total cell lysates (Input) were then analyzed by SDS-PAGE and immunoblotting techniques. Transferred proteins were probed with monoclonal anti-HA or anti-M2 Ab. Molecular weight markers (kDa) are shown to the left. **A**. **Top**: Mature glycosylated α2A and α2C ARs (thick arrow) were predominantly precipitated by avidin, consistent with selective biotinylation of plasma membrane proteins. Aggregated α2 ARs can be seen at the very op of the blot (*). **Bottom**: No REEPs were precipitated by avidin, demonstrating that they were not present at the plasma membrane, when either expressed with α2 ARs or alone. **B**. **Top**: Analysis of total cell lysates for α2A and α2C ARs demonstrated the presence of both mature (thick arrow) and immature forms (thin arrow). **Bottom**: Immunoblotting of total cell lysates for REEPs is shown, demonstrating strong REEP expression. Representative of three experiments.

Previous immunofluorescent analysis had shown inconsistent membrane or organelle localization [1,3,4,16], however our confocal imaging suggested ER, but not plasma membrane, localization. Therefore, we further delineated REEP1/2/6 membrane localization utilizing corroborative biochemical methods. HEK293A cells were transfected with either α2A or α2C ARs and either REEP1, REEP2, or REEP6, followed by live cell plasma membrane biotinylation. Biotinylated surface proteins were then identified by avidin precipitation and immunoblot analysis (Figure 7). Analysis of α2 ARs revealed biotin labeling and avidin precipitation of the mature glycosylated form of the receptor (plasma membrane), but not the immature glycosylated form of the receptor (intracellular/ER), as described previously [25]. The selective biotinylation of the mature glycosylated α2 AR at the plasma membrane served as a positive control for our biotinylation conditions. However, no biotinylated REEP isoforms were seen, even when expressed without α2 ARs.

**Figure 8:**
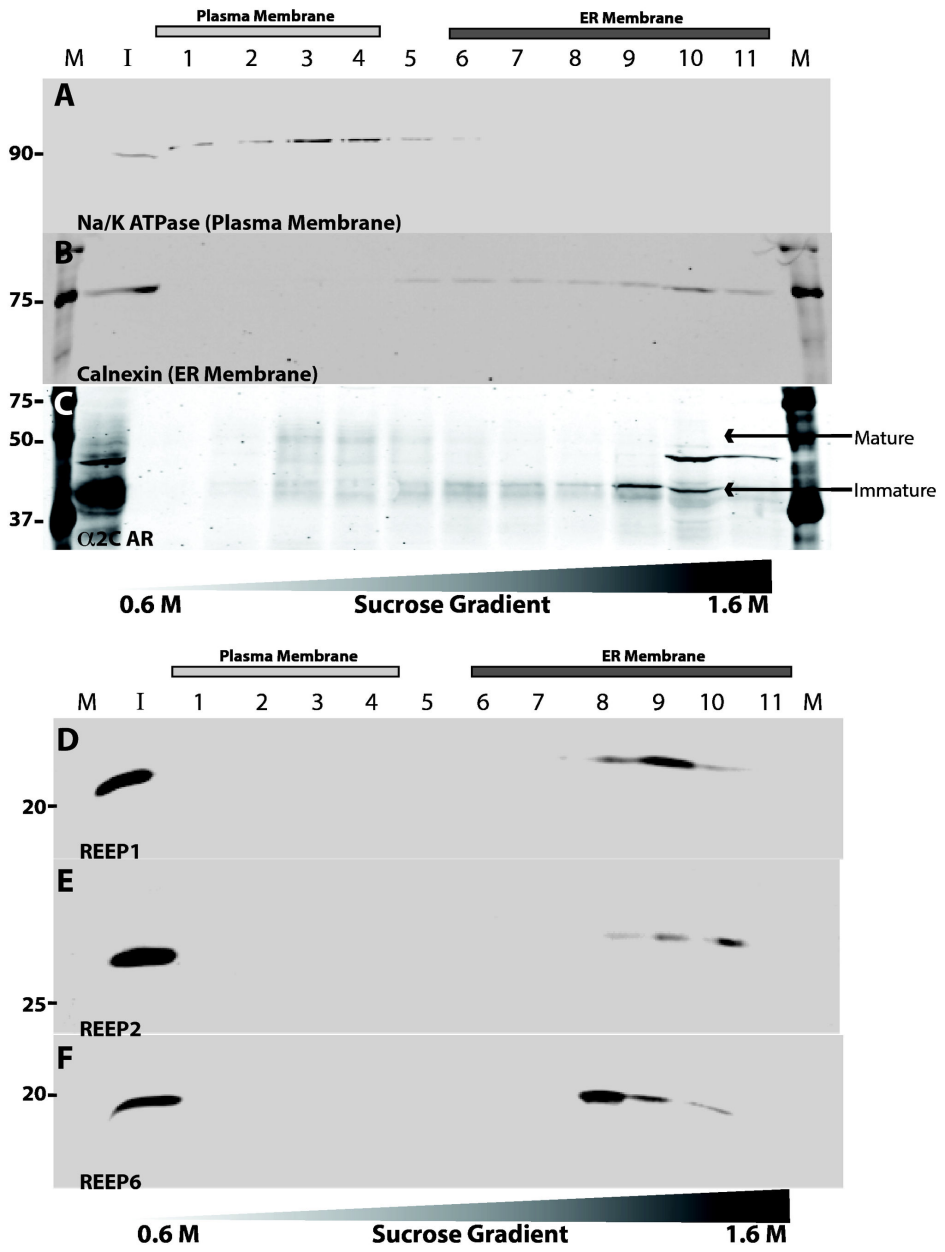
SGMF analysis of REEP membrane localization. To differentiate plasma and ER membrane localization of REEPs, sucrose gradient membrane fractionation (SGMF) analysis was performed with HEK293A cells transfected with HA-α2C ARs, and Flag-REEP1, -REEP2, or -REEP6. Forty-eight hrs post-transfection, total cell membranes were isolated and separated by layering upon a discontinuous sucrose gradient. Eleven fractions were collected and analyzed by SDS-PAGE and immunoblotting techniques. **Top**. Plasma and ER membrane fractions were detected by probing with antibodies against Na/K ATPase (**A**) or calnexin (**B**) respectively. Fractions containing lighter plasma membranes (#1-4) and heavier ER membranes (#6-11) are demarcated with bars above the fraction number. Molecular weight markers (**M**) and input loading control lanes (**I**) were also included. Note progression of α2C ARs (**C**) from ER to plasma membrane fractions, demonstrating increasing mature glycosylation in plasma membrane and decreasing immature glycosylation in ER membrane fractions (see Figure 10). **Bottom**. Similar SGMF analysis of REEP1 (**D**), REEP2 (**E**), and REEP6 (**F**) demonstrating that all three REEPs tested were only found in heavier ER, but not plasma, membrane fractions. Mr standards (kDa) are indicated. Representative of three separate transfections.

To further demonstrate ER localization of REEP proteins, we performed sucrose gradient membrane fractionation (SGMF) analysis. SGMF allows for separation of plasma and ER membranes, thus biochemical differentiation of protein localization. Following transfection of HEK293A cells with α2C ARs and REEP1/2/6, crude membranes were fractionated and aliquots were analyzed by specific antisera to either Na/K ATPase or calnexin, to identify plasma and ER membrane fractions respectively. All REEPs tested showed similar ER membrane fractionation with no expression seen in plasma membrane fractions (Figure 8). The presence of α2C ARs did not alter REEP membrane localization, since expression of REEP1/2/6 alone did not demonstrate plasma membrane localization (data not shown). Therefore, members of both REEP subfamilies reside in the ER and do not appear to traffic to or reside at the plasma membrane at detectable levels in our cell biological and biochemical assays. These results are consistent with our prior confocal microscopy results, further confirming that REEP1/2/6 are not localized to the plasma membrane.

### REEPs do not specifically enhance plasma membrane GPCR expression

Initial analysis of REEP effects on GPCR trafficking utilized immunocytochemical methods, which demonstrated that co-expression of ORs or TRs with REEPs led to enhanced immunofluorescent staining (expression) of GPCRs at the plasma membrane or increased functional expression [1,2]. However, the enhancement of expression observed might represent a true increase in GPCR plasma membrane insertion relative to intracellular GPCR levels (increased plasma membrane/intracellular GPCR ratio = enhanced trafficking) or merely a generalized increase in both plasma membrane and intracellular GPCR levels (unchanged plasma membrane/intracellular GPCR ratio = unchanged trafficking). Previously it had been demonstrated that when α2C ARs are expressed in HEK293A cells, they have a predominant ER localization with minimal plasma membrane expression, whereas α2A ARs efficiently traffic to the plasma membrane [22,25,26]. Given the limitations of immunofluorescent staining for determining the amount of GPCR expressed, we next utilized more quantitative methods to assess the effects of REEP1/2/6 on α2A and α2C AR plasma membrane and intracellular expression.

Previous analysis of REEP effects on GPCR plasma membrane expression or functional expression relied on indirect assays of GPCR activation by ligand (e.g. calcium imaging or luciferase reporter genes) as surrogates for surface expression [1-3]. Though enhancement of cell surface functional activity was noted, it was hypothesized that REEPs enhanced GPCR function by increasing plasma membrane expression specifically. Ligand binding is commonly used to quantify GPCR expression and has not been utilized for the analysis of REEP effects on GPCR expression. Therefore, to further analyze REEP1/2/6 affects on α2 AR expression, [^3^H]RX821002 ligand binding was performed on crude membranes, representing all intracellular and plasma membranes, prepared from cells expressing α2 ARs with and without REEPs (Table 2). Interestingly, co-expression of REEP1/2/6 decreased total membrane binding of α2A ARs. However, α2C AR ligand binding showed a small increase of approximately 20-25% upon co-expression of REEP1 and REEP2. Of note, REEP6 demonstrated a decrease in total membrane binding of α2C ARs, as also seen with α2A ARs. Therefore, REEP1 and REEP2 demonstrated enhancement of α2 AR expression by ligand binding, but the result was α2 AR subtype specific. However, ligand binding of crude membrane preparations cannot delineate intracellular from plasma membrane expression and thus cannot be used to determine the ratio of plasma membrane to intracellular expression of GPCRs (i.e. trafficking) and any possible REEP effects on this ratio.

**Table 2:** Ligand binding analysis of REEP effect on α2 AR expression.

	**Total Binding (%)**
	**Average (± SEM)**
**α2A + Vec**	1.00
**α2A + REEP1**	0.88 (± 0.11)
**α2A + REEP2**	0.73 (± 0.07)
**α2A + REEP6**	0.77 (± 0.16)
**α2C + Vec**	1.00
**α2C + REEP1**	1.24 (± 0.14)
**α2C + REEP2**	1.21 (± 0.19)
**α2C + REEP6**	0.87 (± 0.17)

HEK293A cells were co-transfected with HA-α2A or – α2C ARs and either control vector, Flag-REEP1, - REEP2, or – REEP6 cDNAs. Ligand binding was performed on crude membrane preparations, forty-eight hrs post-transfection. Specific binding of [^3^H]RX-821002 was calculated for each transfection and normalized to α2 AR control binding (n = 5-6).

Ligand binding analysis of co-transfected cDNAs (i.e. GPCRs and REEPs) represents an average of the cell population and presumes that all transfected cells express both cDNAs and that expression of one cDNA does not adversely affect the other, with either possibility skewing the population data. If co-transfection of REEPs and α2 AR cDNAs led to expression in different cell populations, then ligand binding would not accurately reflect REEP effects on α2 AR expression. For example, if α2 ARs were expressed in a higher percentage of cells than REEPs, then analysis of α2 AR expression by ligand binding would include α2 AR-expressing cells that did not express REEPs, thus diluting any possible REEP affects on GPCR expression. To quantify possible differences between REEP and α2 AR expression efficiencies, we performed single cell FACS analysis of transfected cells. By FACS analysis, only 20-35% of cells expressed detectable levels of α2A or α2C ARs, whereas co-expressed REEP1/2/6 proteins were only detectable in 10-30% of cells expressing α2A or α2C ARs (Table 3A). Therefore, transfection of HEK293A cells with REEPs and α2 ARs led to a mixture of cDNA expressing cells, potentially including populations expressing only REEPs, only α2 ARs, both, or neither. Since there exists a mixture of cells expressing various combinations of transfected cDNAs, analysis of the total population of cells by ligand binding or any other population-based cellular assay would possibly lead to inaccurate interpretation of data. Even though there was an effect of REEPs on α2 AR expression, ligand binding did not allow for an analysis of trafficking or determination of a mechanism of action.

**Table 3:** FACS analysis of REEP expression.

**A. Percent α2 AR expressing cells co-expressing REEPs**			
	**+ REEP1**	**+ REEP2**	**+ REEP6**
**α2A AR**	26.6 ± 2.4	29.8 ± 3.1	10.2 ± 1.6
**α2C AR**	36.2 ± 3.7	31.6 ± 2.8	15.3 ± 2.8
**B. REEP expression under non-permeabilized and permeabilized conditions**			
	**Non-**
	**Permeabilized**	**Permeabilized**	**p value**
**%REEP Positive**	4.70 ± 0.44	4.43 ± 0.37	p = 0.64

HEK293A cells were co-transfected with either HA-α2A or - α2C ARs and control vector, Flag-REEP1, - REEP2, or - REEP6 cDNAs. **A**. The total cell population was examined by FACS for α2 AR expression. The α2 AR positive population of cells was then gated for REEP co-expression. The percent of α2 AR positive cells co-expressing REEP is shown (± SEM). The overall expression efficiency for α2 ARs ranged from 20-35% of cells transfected (data not shown). REEPs were co-expressed in approximately 10-36% of α2 AR positive cells (n = 4-8). **B**. The total cell population was examined by FACS for REEP expression under non-permeabilized and permeabilized conditions. The results for REEP1, REEP2, and REEP6 were combined and the percent of cells expressing REEPs is shown (± SEM) for each condition (n = 41). Data was analyzed by an unpaired Student t-test. A similar percentage of cells expressed REEPs under both sets of conditions, demonstrating that the use of non-permeabilized cells for FACS analysis identified the same population of REEP-expressing cells as seen with permeabilization.

### Quantitative FACS analysis of REEPs and GPCRs

FACS analysis is unique in that it can assay individual cells based upon immunofluorescent labeling and then quantify the intensity of the labeling to allow for relative measurements of protein expression. Additionally, expression of multiple proteins can be performed at the same time, on the same cell, by use of different fluorescent-conjugated antibodies, making FACS a powerful cell biological assay. For example, FACS has been utilized to calculate the ratio of GPCR plasma membrane to intracellular expression, in order to examine GPCR trafficking motifs [23]. Similarly, FACS would be a more accurate measure of plasma membrane expression and would more clearly determine if REEPs specifically enhance GPCR plasma membrane expression. Given the above findings concerning differential REEP/α2 AR expression following transfection, a single cell analysis method would allow for a separation of these various cell populations and for study of only cells that express both REEPs and α2 ARs, negating the need for complex transfection strategies (e.g. polycistronic cDNA constructs). Thus, we studied REEP modulation of GPCR expression at the single cell level, utilizing a FACS assay we developed to measure surface vs. total GPCR expression for analysis of GPCR trafficking motifs [25].

In order to properly quantify the ratio of α2 AR surface and intracellular receptor expression with FACS analysis and then examine the effect of co-expressed REEP proteins, immunofluorescent labeling of transfected cells must be performed under permeabilized and non-permeabilized conditions. Given that REEPs are not found on the plasma membrane, a method for identifying REEP-expressing cells under non-permeabilized conditions was needed. It has been shown previously [27], that antibodies can gain intracellular entry into PFA-fixed cells under a variety of conditions and thus label cytoplasmic protein epitopes, such as carboxyl terminus Flag epitope-tagged REEPs (Figure 1).

**Figure 9:**
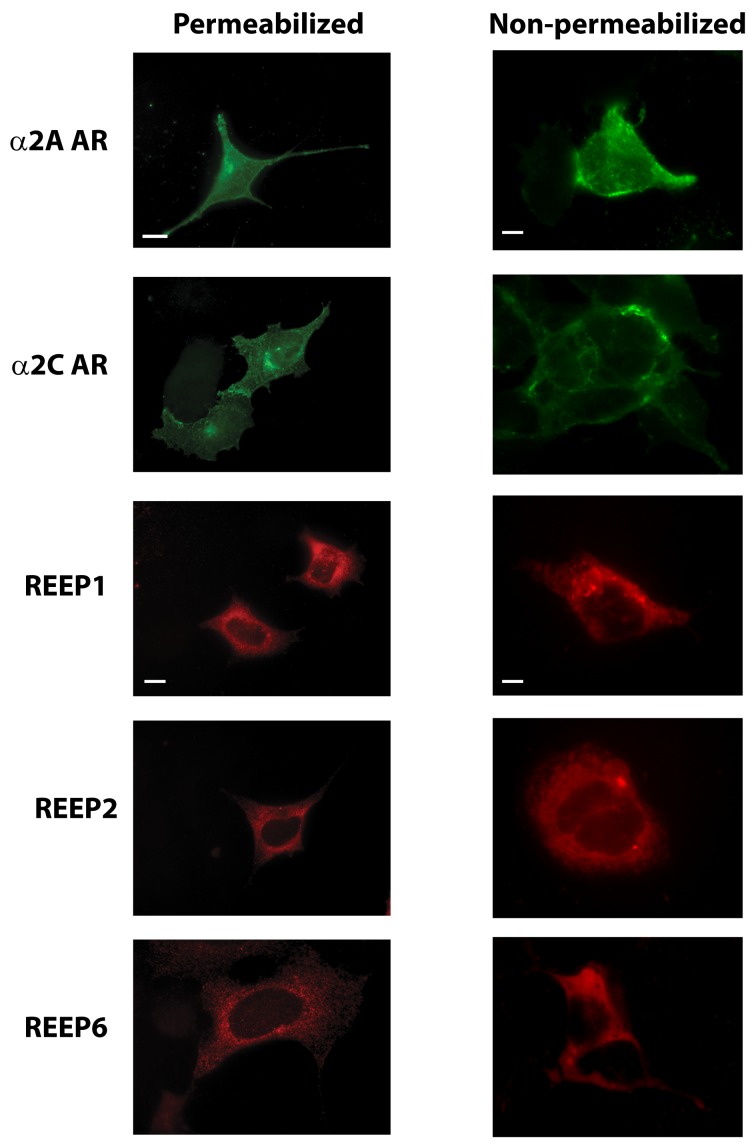
Identification of α2 AR and REEP expression in permeabilized and non-permeabilized cells. HEK293A cells were transfected with HA-α2A AR, HA-α2C AR, Flag-REEP1, Flag-REEP2, or Flag–REEP6 cDNA. Forty-eight hrs post-transfections, cells were fixed with 4% PFA, under permeabilized and non-permeabilized conditions, and examined by wide field immunofluorescent microscopy. HA-α2 ARs were labeled with 16B12-conjugated FITC (anti-HA) antisera and Flag-REEPs were identified by M2-conjugated Alexa 594 (anti-Flag) antisera. **Permeabilized**: α2 AR staining of permeabilized cells demonstrated plasma membrane and intracellular fluorescence. Note predominant plasma membrane staining, compared to intracellular staining, consistent with efficient plasma membrane trafficking of α2A ARs. α2C AR staining revealed predominant intracellular staining with a perinuclear shadow, due their predominant localization within the ER when expressed in HEK293A cells (25). REEP staining of permeabilized cells showed strong intracellular localization, due to their ER localization. **Non-permeabilized**: α2A ARs demonstrated extensive staining of the plasma membrane, however, α2C ARs showed staining only of the plasma membrane, demonstrating that the extracellular HA epitope was not accessible by anti-HA antibody under non-permeabilized conditions. Intracellular REEP staining was similar when performed under permeabilized and non-permeabilized conditions, demonstrating that the carboxyl terminal Flag epitope was accessible by anti-Flag antibody under either condition, due to its cytoplasmic localization. REEP staining of non-permeabilized cells revealed intracellular staining, due to the ability of antibodies to gain intracellular entry following PFA fixation [27]. Representative of three separate transfections. Scale bars: 10 µm.

To further clarify this method, we transfected HEK293A cells with HA-α2 ARs or Flag-REEP1/2/6 and examined their expression in PFA fixed, but non-permeabilized, cells using primary conjugated antibodies to mimic the FACS methodology (Figure 9). Wide-field microscopy was used in order to allow for a larger field of cells for study. As seen in Figures 1 and 6, apparent intracellular staining of Flag-REEP1/2/6 was seen despite the lack of permeabilization, as evidenced by the immunofluorescent identification of a fine trabecular network originating from a perinuclear shadow. Intracellular staining without permeabilization was possible because the Flag epitope is localized to the cytoplasmic space and thus is accessible after PFA fixation [27]. However, no intracellular labeling was seen for α2 ARs since the HA epitope is located on the extracellular amino terminus of the receptor, which would be localized to the ER intraluminal space inside of cells. Without permeabilization, antibodies cannot efficiently gain access across this second membrane compartment, thus preventing intracellular labeling of α2 ARs under non-permeabilized conditions. The differential ability to immunofluorescently label and identify REEP-expressing cells in the absence of permeabilization, while maintaining the ability to localize only plasma membrane expression of α2 ARs in these same non-permeabilized cells, allowed for quantitative FACS analysis of REEP-effects on GPCR trafficking.

**Figure 10:**
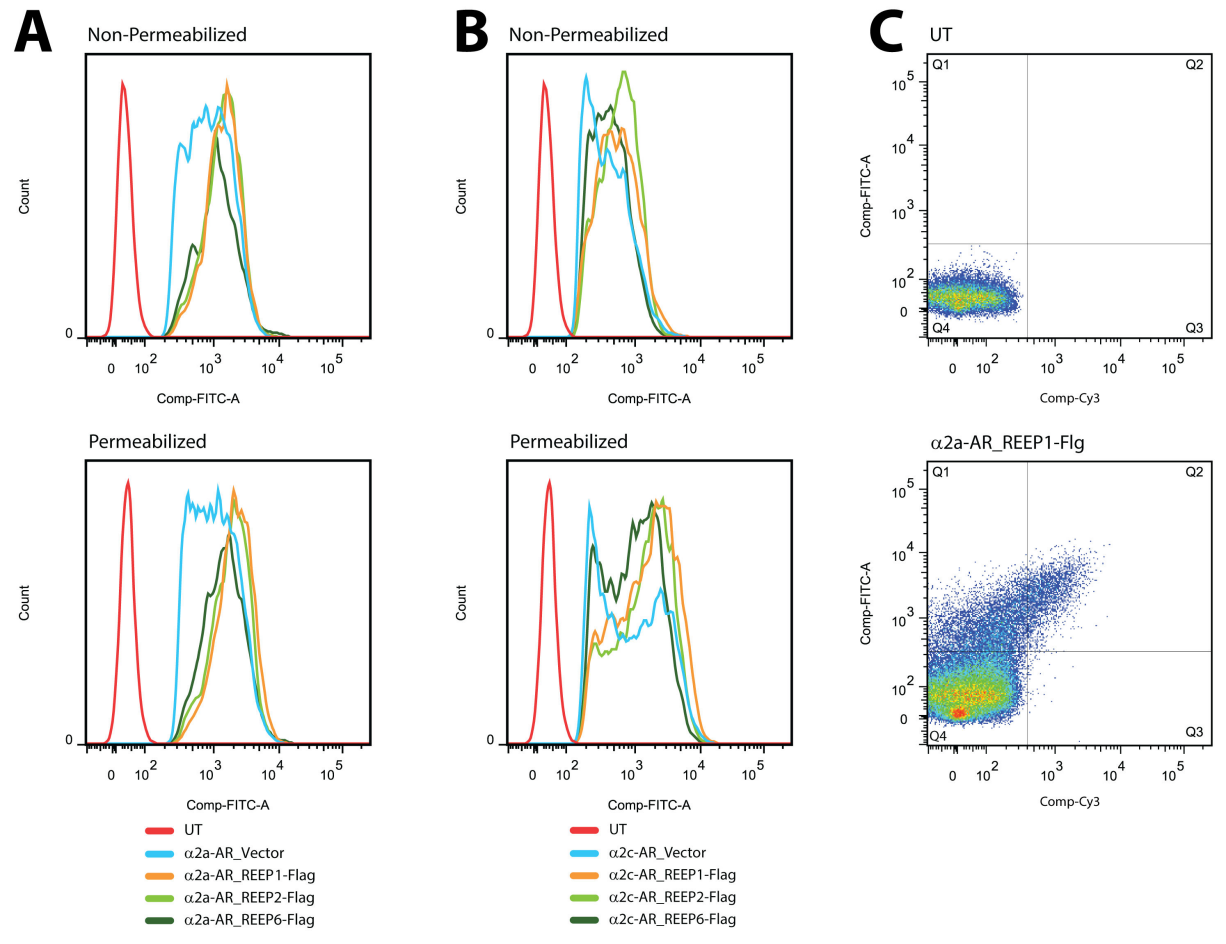
Representative FACS histograms and REEP gating strategy. HEK293A cells were co-transfected with HA-α2A or -α2C ARs and control vector, Flag-REEP1, -REEP2, or –REEP6 cDNAs. Forty-eight hrs post-transfection, relative expression levels of each receptor were determined in non-permeabilized (Surface) and permeabilized (Total) cells by using a FACS assay. α2 ARs and REEPs were labeled with FITC-conjugated anti-HA and Cy3-conjugated anti-Flag (M2) antibodies respectively. **A**. Representative α2A AR FACS fluorescence distributions under non-permeabilized (Top) and permeabilized (Bottom) conditions (UT = untransfected). **B**. Representative α2C AR FACS fluorescence distributions under non-permeabilized (Top) and permeabilized (Bottom) conditions (UT = untransfected). Note the shift to higher median fluorescence upon permeabilization, which is greater for α2C vs. α2A ARs due to the larger pool of intracellular α2C ARs [25]. **C**. Representative gating strategy for FACS analysis of co-expressed α2 ARs and REEPs. Background staining of non-transfected HEK293A cells with FITC-conjugated anti-HA and Cy3-conjugated anti-Flag (M2) antibodies was determined (Top) and used to set the FACS gating thresholds for background fluorescence (Q4). Representative α2A AR and REEP1 FACS data set demonstrating co-expression of both proteins is shown (Bottom). All cells contained in quadrants Q1-3 were analyzed for calculation of REEP effects on co-expressed α2 AR surface and intracellular expression (see Table **4A**). Data summarized from between five and eight different transfections for each combination of α2 AR and REEP with a minimum of 1000 cells analyzed for each transfection.

To quantify REEP effects on α2 AR trafficking by FACS, HEK293A cells were co-transfected with various combinations of REEP1/2/6 and α2A or α2C ARs. For further analysis of REEP effects on α2 AR expression, selective gating of cells expressing both REEPs and α2 ARs were analyzed to determine the percentage of surface and intracellular GPCR expression. First, the cell population was divided, and subsequently examined for α2 AR expression under non-permeabilized (surface) and permeabilized (total) conditions. As can be seen in representative FACS histograms (Figure 10A/B), α2A AR-expressing cells exhibit a higher median fluorescent intensity in non-permeabilized cells (surface expression), compared to α2C ARs. This finding is consistent with our prior data demonstrating a higher level of α2A AR plasma membrane expression in HEK293A cells [25]. However, following permeabilization (total expression), all α2A and α2C AR expressing cells demonstrate a shift towards higher median fluorescent intensities. This affect is more pronounced for α2C AR expressing cells (Figure 10B), since they have a larger intracellular pool of receptor due to ER retention [25]. Co-expression of REEP1/2/6 demonstrated a similar shift in α2 AR median fluorescent intensity following permeabilization (Figure 10A/B). Note that the histograms demonstrate a heterogeneous population of α2 AR expressing cells as measured by median fluorescent intensity, as described previously [25].

The ability of Flag-tagged REEP1/2/6 to be detected by immunofluorescence in non-permeabilized (Figure 9) and permeabilized cells allowed for identification of REEP expressing cells under both conditions, thus allowing for complete FACS analysis of α2 ARs in cells that were non-permeabilized (plasma membrane expression) or permeabilized (total expression). Detection of Flag-tagged REEPs under both conditions was similar (Table 3B), demonstrating that this strategy identified similar population of REEP-expressing cells for subsequent α2 AR analysis. To determine the effect of REEP1/2/6 co-expression on α2A and α2C ARs, single cells were gated by FACS for the expression of REEPs, and subsequently assayed for α2 AR expression, as measured by median fluorescent intensity of the selected cell population. A representative gating strategy is provided (Figure 10C). Fluorescent intensity (arbitrary units) for REEP (Cy3, x-axis) and α2 AR (FITC, y-axis) is shown on a FACS dot plot. The top panel represents untransfected cells, which were utilized to detect background FITC/Cy3 immunofluorescence and thus set FITC/Cy3 gating conditions. The bottom panel shows a representative dot plot of HEK293A cells expressing α2A ARs and REEP1. Note the shift in median fluorescent intensity of α2A ARs upon REEP1 co-expression, as demonstrated in quadrant Q2 compared to Q1. Also, note that there is a continuum of REEP fluorescent intensities above a threshold set by our gating strategy, and thus all REEP-positive cells were analyzed, not only high REEP-expressing cells.

A multi-step process was required to perform a complete FACS analysis of α2 AR expression. First, live/dead cell populations were identified using Live/Dead Fixable Violet™ stain and only the live cell population was identified and studied by FACS for analysis of REEP and α2 AR expression. Following gating for REEP-expressing cells under permeabilized and non-permeabilized conditions; we next measured the median fluorescence of surface and total α2 ARs within this REEP-expressing population. Because α2A and α2C ARs express at different levels, analysis of median fluorescent intensities alone would not allow for a direct comparison of REEP effects on these two GPCRs. Therefore, we calculated the percentage of plasma membrane (surface) and intracellular expression of α2 ARs in order to normalize the two receptor populations and subsequent effects of REEP co-expression (Table 4A), as described previously [23].

**Table 4:** FACS analysis of REEP effect on α2 AR expression.

**A. Gated - REEP**				
	α**2A AR**	α**2A AR**	α**2C AR**	α**2C AR**
	**% Surface**	**% Intracellular**	**% Surface**	**% Intracellular**
	**Expression**	**Expression**	**Expression**	**Expression**
**Control**	81.8 ± 2.4	18.2 ± 2.4	42.3 ± 2.5	57.7 ± 2.5
**+ REEP1**	59.1 ± 3.3	40.9 ± 3.3	34.7 ± 3.4	65.3 ± 3.4
**+ REEP2**	63.0 ± 2.1	37.0 ± 2.1	34.7 ± 2.5	65.3 ± 2.5
**+ REEP6**	53.3 ± 2.5	40.3 ± 2.5	40.3 ± 2.3	59.7 ± 2.3
**B. Not Gated − REEP**				
	α**2A AR**	α**2A AR**	α**2C AR**	α**2C AR**
	**% Surface**	**% Intracellular**	**% Surface**	**% Intracellular**
	**Expression**	**Expression**	**Expression**	**Expression**
**Control**	81.8 ± 2.4	18.2 ± 2.4	42.3 ± 2.5	57.7 ± 2.5
**+ REEP1**	80.8 ± 3.0	19.1 ± 3.0	51.7 ± 3.0	48.3 ± 3.0
**+ REEP2**	75.9 ± 3.8	24.1 ± 3.8	43.8 ± 2.5	56.2 ± 2.5
**+ REEP6**	77.9 ± 4.0	22.1 ± 4.0	55.1 ± 2.6	44.9 ± 2.6
**C. Change in Expression (Gated – REEP)**				
	α**2A AR**	α**2A AR**	α**2C AR**	α**2C AR**
	**Surface**	**Total**	**Surface**	**Total**
	**Expression**	**Expression**	**Expression**	**Expression**
**Control**	1.00	1.00	1.00	1.00
**+ REEP1**	1.96 ± 0.12	2.69 ± 0.24	1.58 ± 0.14	1.96 ± 0.12
**+ REEP2**	2.06 ± 0.15	2.61 ± 0.20	1.70 ± 0.18	2.07 ± 0.14
**+ REEP6**	1.34 ± 0.17	1.99 ± 0.19	1.46 ± 0.15	1.48 ± 0.16

**A**. Following gating for REEP expressing cells only, median fluorescence values for α2A and α2C ARs were measured in permeabilized and non-permeabilized cells under each transfection condition and the percent surface and intracellular expression of α2 ARs were calculated (± SEM). Mock vector (no REEP) was used for control (these cells were not gated for REEP expression). **B**. Reanalysis of the same data set without gating for REEP expressing cells demonstrates no change in surface or intracellular expression of α2A and α2C ARs by REEP co-expression. Mock vector (no REEP) was used for control. **C**. The change in α2 AR expression when co-expressed with REEPs were determined by measuring median fluorescent intensity of surface (non-permeabilized) and total (permeabilized) α2 ARs and calculated as fold-induction (± SEM). Cells were gated for REEP expression prior to analysis. Mock vector (no REEP) was used for control. Note that REEP co-expression enhanced both surface and total α2 AR expression regardless of α2 AR subtype. A minimum of 1000 REEP expressing cells were used from each transfection and data represents the average of 4-8 experiments for each condition.

FACS analysis revealed no enhancement for any combination of REEP or α2 AR tested. In fact, REEP co-expression decreased the percentage of α2A ARs expressed at the plasma membrane (81.8% to approximately 60%) and increased intracellular levels of α2A ARs. However, REEP co-expression did not drastically alter α2C AR plasma membrane expression (approximately 35-40% with or without REEP). Thus REEP co-expression did not enhance the plasma membrane/intracellular ratio of either α2 ARs, thus demonstrating no specific enhancement of GPCR trafficking to the cell surface.

To determine the relative importance of gating for REEP prior to analysis of α2 AR expression, the same single cell data set was used to calculate plasma membrane and intracellular α2 AR membrane expression irrespective of REEP expression (Table 4B). As can be seen, this analysis demonstrated no effect of REEP1/2/6 on the relative ratios of plasma membrane and intracellular expression of either α2A or α2C ARs. By not selecting REEP expressing cells prior to α2 AR FACS analysis, the data was skewed and REEP effects were not observed, thus demonstrating the importance of multi-channel single cell FACS analysis.

Prior research identified REEPs by their ability to enhance functional OR plasma membrane expression [1]. The median fluorescent intensities of α2 ARs increased upon co-expression with REEPs (data not shown), consistent with an increase in total α2 AR protein production. Thus, we calculated the relative increase in plasma membrane (non-permeabilized) and total (permeabilized) α2 AR expression in REEP-positive cells, revealing that REEP co-expression enhanced the amount of both plasma membrane and total α2A and α2C AR expression (Table 4C). Taken together, these data suggests that REEP1/2/6 co-expression enhanced cargo capacity of the ER (intracellular expression), but did not specifically enhance plasma membrane expression of α2A or α2C ARs. Therefore, the previously described REEP enhancement of plasma membrane GPCR expression seen by immunocytochemistry and inferred by functional receptor screening [1-3] may likely represent an increase in both total and plasma membrane receptor expression and not a specific REEP affect on GPCR trafficking.

### REEP co-expression alters α2C glycosylation

α2 ARs undergo a variety of maturation steps (e.g. glycosylation) as they traffic from ER to plasma membranes, which can be monitored utilizing biochemical methods [25]. Though REEP1/2/6 did not demonstrate selective enhancement of GPCR plasma membrane expression, they did show an overall enhancement of total receptor levels and ER cargo capacity. Previous work with other Yip family members has demonstrated alterations in ER retention and glycosidic processing [33]. To further assess REEP effects on intracellular cargo protein processing, α2 ARs and REEP1/2/6 were co-expressed and membrane fractions were isolated and analyzed by use of specific endoglycosidases.

**Figure 11:**
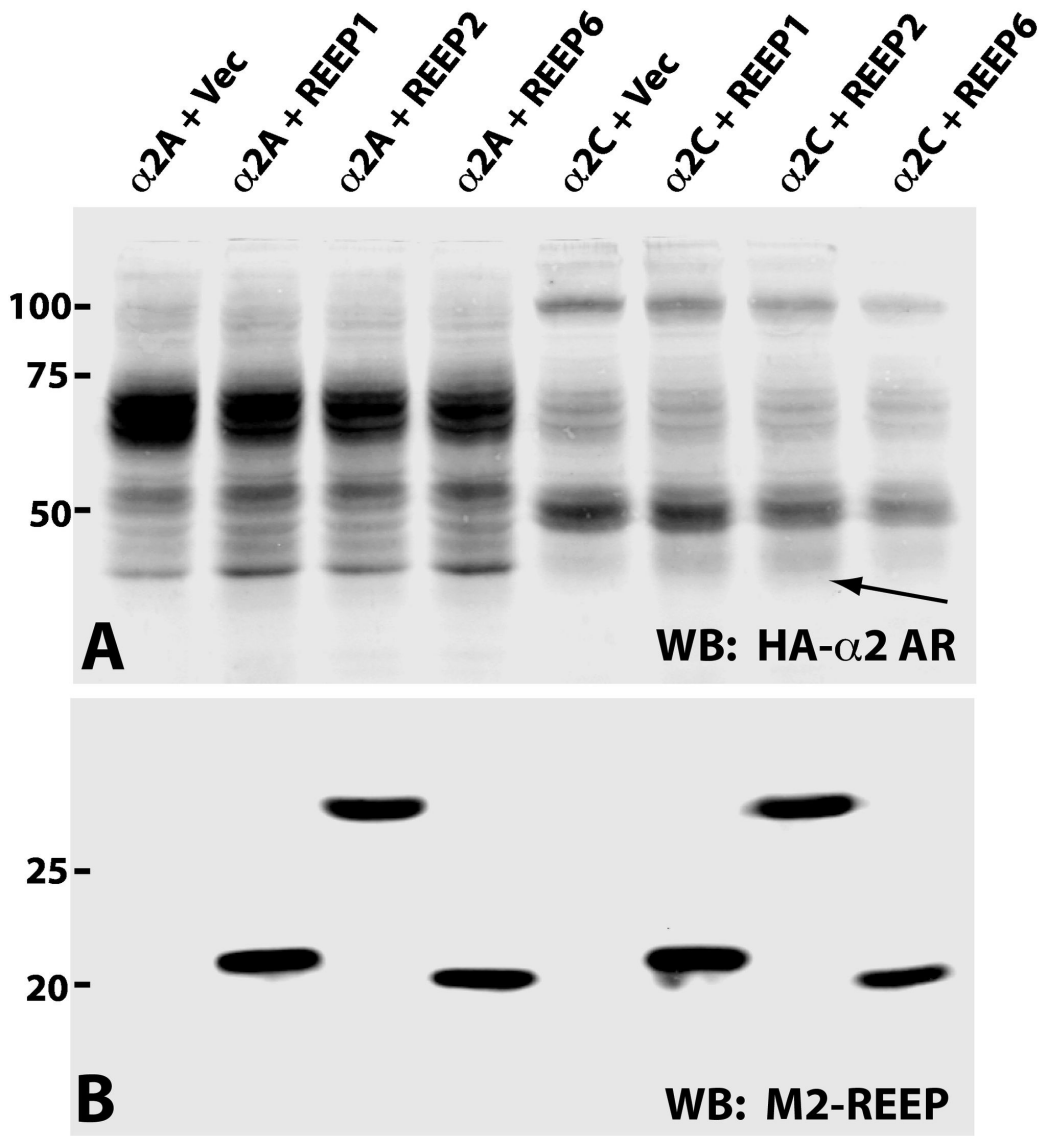
REEP co-expression enhances the presence of a lower molecular weight form of α2C ARs. HEK293A cells were transfected with either HA-α2A or -α2C ARs and control vector, Flag-REEP1, -REEP2, or –REEP6. Forty-eight hrs post-transfection, crude membranes were isolated and subjected to immunoblot analysis. Molecular weight markers (kDa) are shown to the left. **Top**: Co-expression of either REEP1, REEP2, or REEP6 with α2C AR correlated with an increased detection of a lower molecular weight form of α2C AR (arrow), not seen following co-expression with α2A ARs. **Bottom**: Immunoblot analysis of REEPs demonstrated similar levels of REEP1, REEP2, and REEP6 expression when co-expressed with either α2A or α2C ARs. Representative of three experiments.

In HEK293A cells, α2A ARs show primarily a mature glycosylation pattern consistent with plasma membrane expression; α2C ARs show predominantly immature glycosylation due to ER retention (Figure 11), as described previously [25]. Analysis of α2 ARs co-transfected with REEP1/2/6 demonstrated that REEP co-expression did not appear to alter the appearance of mature and immature α2A or α2C ARs. However, co-expression of either REEP1/2/6 led to an increase in a lower molecular weight form of α2C ARs that was not seen with α2A ARs. Interestingly, this lower molecular weight form is evident in the heavier ER membranes from the SGMF analysis (Figure 8, fractions 9 and 10).

**Figure 12:**
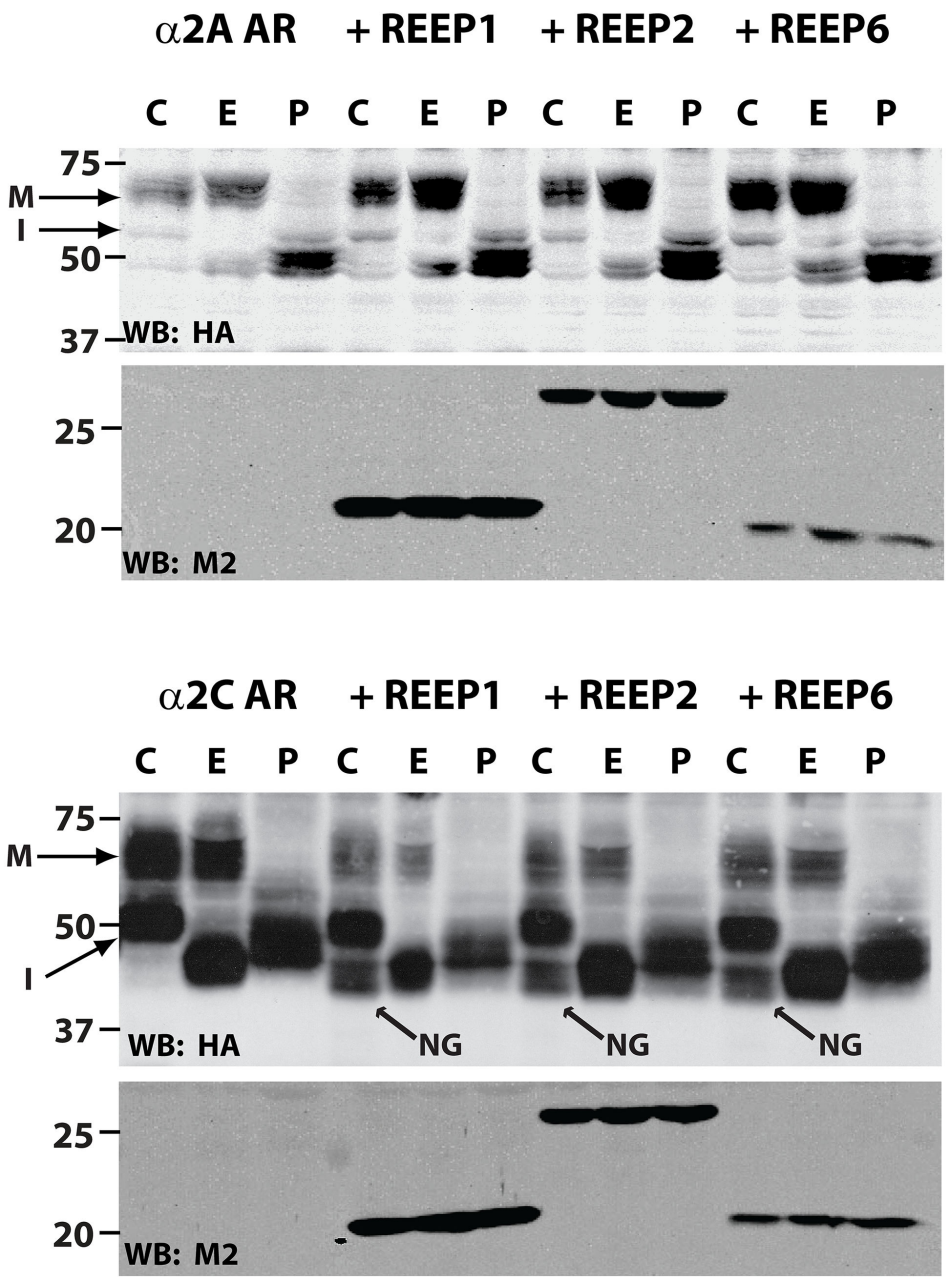
REEP co-expression enhances the presence of a minimally-glycosylated form of α2C ARs. HEK293A cells were transfected with either HA-α2A or -α2C ARs and control vector, Flag-REEP1, -REEP2, or –REEP6. Forty-eight hrs post-transfection, crude membranes were isolated and subjected to endoglycosidase digestion (**C** = No Enzyme, **E** = Endoglycosidase H (Endo H), **P** = PNGase F (PNGase)). Due to loss of signal during enzymatic digestion, 125 µg of protein was digested and loaded in each lane. Mature (**M**) and immature (**I**) glycosylated forms are indicated. Endo H cleaves only immature glycosylated forms, whereas PNGase cleaves all glycosylated forms. Molecular weight markers (kDa) are shown on the left. Note that α2A ARs exhibit mostly mature (Endo H insensitive), whereas α2C ARs show mostly immature (Endo H sensitive), glycosylation patterns, correlating with their predominant plasma membrane and intracellular localizations respectively. The presence of REEP1, REEP2, or REEP6 with either α2 AR did not alter the relative ratios of mature to immature glycosylation. However, co-expression of either REEP1, REEP2, or REEP6 with α2C ARs correlated with an increased expression of a lower molecular weight form that was minimally glycosylated (arrow), not seen following co-expression with α2A ARs. Apparent loss of the minimally glycosylated form after PNGase treatment was due to instability in PNGase enzymatic buffer conditions (data not shown). Representative of three experiments.

To further assess for possible REEP effects on α2A and α2C AR maturation, membrane fractions from co-transfected cells were digested with Endoglycosidase H (Endo H) and Peptide: N-Glycosidase F (PNGase). Endo H cleaves only immature N-linked glycans (ER resident), whereas PNGase cleaves both mature and immature N-linked glycans. Endoglycosidase analysis of α2 ARs co-transfected with REEP1/2/6 demonstrated that REEP co-expression did affect the overall maturation and trafficking of α2C ARs (Figure 12). However, the lower molecular weight form of α2C ARs seen when co-expressed with REEP1/2/6 was insensitive to Endo H deglycosylation. Given that Endo H can cleave only immature glycans, this new lower molecular weight form most likely represents a minimally or non-glycosylated form of the receptor and not a degradation product. If this lower molecular weight form was due to degradation of an immature glycosylated form, Endo H digestion would have caused a decrease in its molecular weight, which was not observed. Thus, REEP1/2/6 co-expression can lead to specific alterations in cargo glycosylation, possibly due to changes in ER/Golgi processing or retention. More importantly, this data suggests that REEP1/2/6 can differentially modulate intracellular processing of two highly homologous receptor proteins.

### REEPs can selectively interact with GPCR cargo

**Figure 13:**
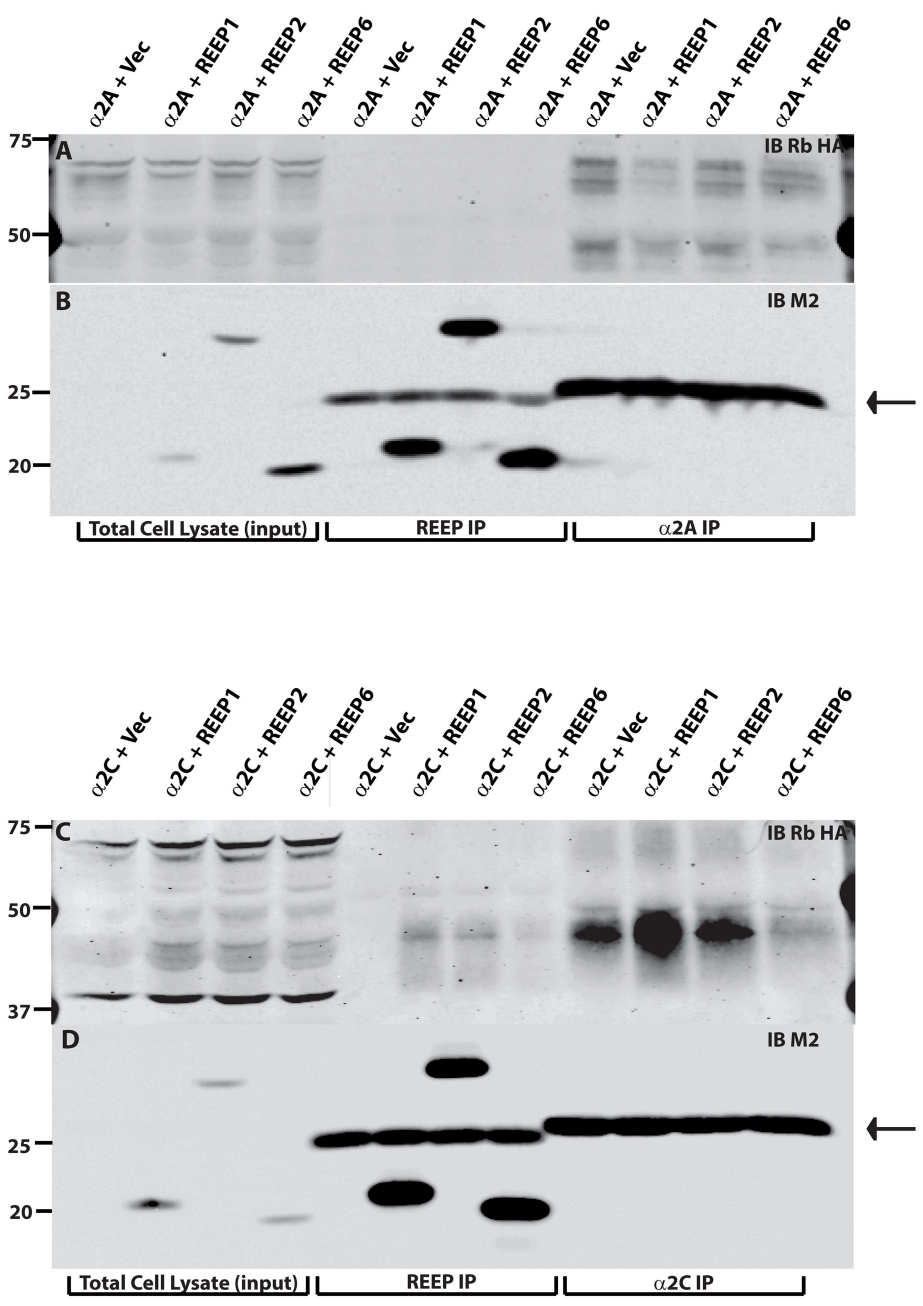
Immunoprecipitation of α2 ARs by REEPs. HEK293A cells were co-transfected with HA-α2A or -α2C ARs and control vector, Flag-REEP1, -REEP2, or –REEP6. Eighteen hrs post-transfection, total cell lysates were isolated and analyzed by co-immunoprecipitation (co-IP) with M2 antibody (anti-Flag) and immunoblotting. Transferred proteins were probed with rabbit anti-HA Ab (A/C) or anti-M2 (B/D). Molecular weight markers (kDa) are shown to the left. Lanes representing total cell lysates (input), REEP co-IP, and α2 AR co-IP are labeled at the bottom of the blots. **A**. Note absence of α2A AR co-IP with any REEP tested (middle). **B**. Similar amounts of REEP1, REEP2, and REEP6 were present in REEP co-IP assays. **C**. REEP1, REEP2, and REEP6 could co-IP the REEP-enhanced, minimally glycosylated form of α2C AR (Figures 9 and 10). **D**. As seen with α2A AR/REEP co-IP assays, similar amounts of REEP1, REEP2, and REEP6 were present in REEP co-IP assays. Neither α2A nor α2C ARs could co-IP any REEP tested (B and D, right). IgG light chain artifacts are indicated by an arrow (far right).

REEP1/2/6 can all enhance the cargo capacity for both α2A and α2C ARs as assayed by FACS analysis (Table 4), though they only appear to alter intracellular trafficking of α2C ARs, as assessed by deglycosylation analysis (Figures 11 and 12). Therefore, co-immunoprecipitation assays were utilized to determine if a specific interaction occurred between REEP1/2/6 and α2 ARs. REEP1/2/6 and α2 ARs were co-transfected into HEK293A cells, and membrane extracts were immunoprecipitated with anti-Flag antibodies (REEP) and recovered proteins were analyzed by immunoblotting (Figure 13). Analysis of co-expressed REEP1/2/6 and α2 ARs demonstrated that only α2C, but not α2A, ARs interacted with REEPs, and most interestingly, the interacting forms of α2C ARs were the previously identified REEP-enhanced, minimally or non-glycosylated forms. Note the absence of mature (plasma membrane) α2C ARs in the co-IP, further suggesting the absence of REEPs at the plasma membrane and thereby being able to interact with α2C ARs. The inability to detect a reverse immunoprecipitation of REEPs by α2C ARs may reflect the relative low-level expression of these α2C AR forms or may reflect steric hindrance from other unidentified proteins. This result suggests that REEPs can specifically identify cargo protein to alter intracellular transport and processing.

### HSP REEP1 mutation affects cargo interactions

**Figure 14:**
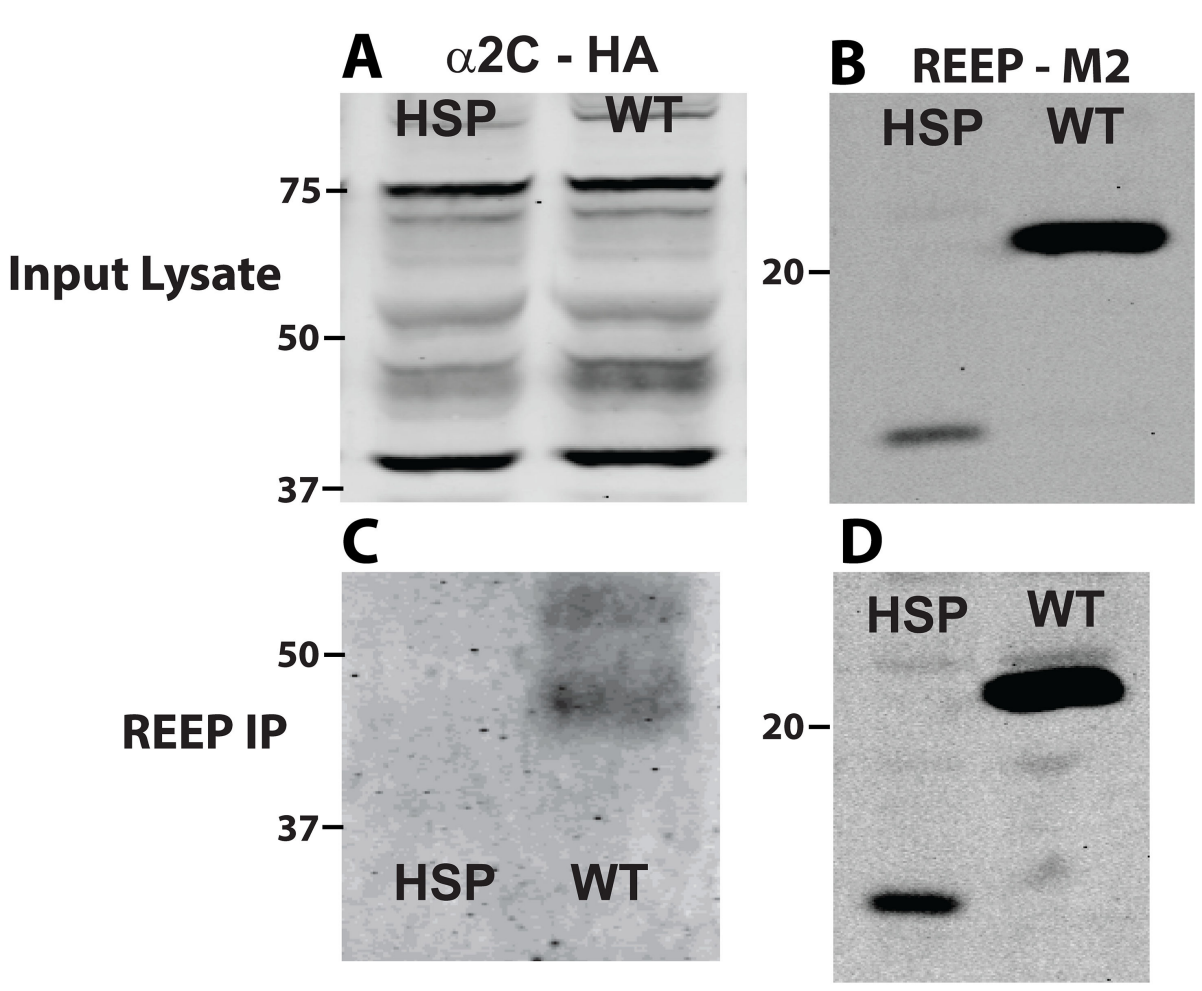
REEP1 HSP mutant Arg113X does not interact with α2C ARs. HEK293A cells were co-transfected with HA-α2C AR and either Flag-REEP1 (WT) or Flag-REEP1 (HSP), an HSP mutant form of REEP1 with a premature stop codon at Arg113, leading to a loss of the carboxyl terminus. Eighteen hrs post-transfection, total cell lysates were isolated and analyzed by co-immunoprecipitation (co-IP) with M2 antibody (anti-Flag) and immunoblotting. Transferred proteins were probed with anti-HA Ab or anti-M2 antibodies. Molecular weight markers (kDa) are shown to the left. Inputted total cell lysates were probed for either α2C ARs (**A**) or REEP (**B**). Note smaller size of HSP mutant REEP1, consistent with the loss of the carboxyl terminus, and lower expression levels seen following transfection. Following REEP co-IP, WT REEP1, but not HSP mutant REEP1, was able to immunoprecipitate the minimally glycosylated form of α2C AR (**C**). REEP co-IP immunoblot is shown (**D**). Representative of three experiments.

The ER membrane shaping properties of REEPs may represent only part of their cellular function, and their ability to interact with cargo and other proteins may represent another function relevant to HSP pathogenesis. Such an interaction between REEPs and potential cargo proteins could occur via the hydrophobic hairpins or the carboxyl terminus. Several HSP-inducing mutations of REEP1 involve premature truncation of the carboxyl terminus, such as REEP1Arg113X [5]. This mutant form involves a single nucleotide polymorphism causing a premature truncation at Arg113 and thus loss of the majority of the carboxyl terminus. Similar co-immunoprecipitation experiments were performed to investigate the role of the carboxyl terminus in REEP1 protein-protein interactions (Figure 14). Surprisingly, deletion of the carboxyl terminus led to a loss of the interaction between REEP1 and α2C ARs. Therefore, REEP1 can alter cargo protein trafficking, via an interaction with its carboxyl terminus.

## Discussion

### REEPs and GPCR Trafficking

Prior work examining the effects of REEP co-expression on ORs and TRs suggested that REEPs “enhanced” cell surface and functional expression, possibly by acting as chaperones or co-receptors [1-3]. Additionally, it has been suggested that REEP2 may aid recruitment of sweet TRs to lipid rafts, thus affecting localization [3]. However, it was noted that REEPs had specificity to their effects and did not enhance expression of all GPCRs. For example, REEP1 enhanced expression of ORs, but not V2R pheromone receptor or ß2 ARs [1]. REEP2 was shown to enhance sweet TRs, T1R and T2R, but not 5-HT_1A_ serotonin receptors nor 5-HT3 ion channels [3]. Lastly, multiple REEPs were shown to enhance ligand responsiveness of co-expressed bitter TRs [2].

The lack of clarity with respect to REEP effects on GPCR expression may be due to the methods utilized, including functional assays of receptor activation by ligand or immunocytochemical staining. Overall, functional assays do not measure actual receptor levels and cannot differentiate between an affect due to increased plasma membrane receptor expression or co-receptor dependency. Immunocytochemical methods do not quantify levels of membrane expression, so we applied more quantitative methods. Ligand binding revealed that some combinations of REEPs had a paradoxical effect on GPCR expression, with REEP1/2 enhancing and REEP6 decreasing α2C AR binding (Table 2). Similar effects of REEP co-expression were observed with bitter TRs, where it was noted that REEP6 co-expression decreased responsiveness to ligand activation, compared to other REEPs [2].

Ligand binding on transfected cells assumes that every transfected cell is expressing both REEPs and α2 ARs. Therefore, attempting to measure the effect of REEP co-expression on a GPCR is not optimal when the population of cells expressing the GPCR is not homogeneous with respect to GPCR and REEP expression, which we observed by FACS analysis (Table 3). Also, if REEPs enhanced GPCR expression by acting as trafficking chaperones, then the ratio of plasma membrane to intracellular expression should increase. In order to test this hypothesis and truly measure REEP effects on GPCR expression, FACS analysis was optimized to analyze only single cells expressing both REEP and α2 ARs (Table 4). This study is the first to our knowledge to assess GPCR plasma membrane expression and correlate it with REEP expression in single cells. Using multi-channel FACS, we were able to demonstrate the REEP1/2/6 do not preferentially enhance plasma membrane expression of either α2A or α2C ARs, instead they increase the overall ER cargo capacity for α2 ARs and thus total α2 AR protein levels. Prior immunocytochemical and functional analyses demonstrated an apparent increase in GPCR expression at the cell surface when co-transfected with REEPs [1], however potential simultaneous increases in intracellular GPCRs were not discerned, thus giving the plausible appearance of a selective trafficking affect of REEPs on GPCRs.

When REEPs were originally identified, it was noted that REEP1 did not preferentially enhance plasma membrane expression of all GPCRs (e.g. ORs vs. ß2 ARs) [1]. We saw a similar affect with α2A and α2C ARs. At steady state, heterologous expression of α2A ARs leads to efficient delivery to the plasma membrane; α2C ARs are more predominantly localized to the ER [20,22,25]. It is possible that transit time through the ER may explain the relative responsiveness of different GPCRs to REEP modulation or interactions. For example, the longer ER resident time for heterologously expressed α2C ARs could allow for more interaction time with REEPs, as demonstrated by immunoprecipitation, possibly accounting for the increased presence of the minimally glycosylated form. Alternatively, trafficking of α2A and ß2 ARs from the ER may be operating at maximal transport efficiency and thus increasing ER cargo capacity by REEP co-expression may not alter plasma membrane expression, thus leading to ER retention as noted by FACS analysis (Table 4) [22]. Thus, innate dissimilarities in intracellular trafficking efficiencies for various GPCRs may account for the differential enhancement of cell surface expression seen upon REEP co-expression, as seen by others and us. A third possibility is that REEPs interact with different ER-generated vesicles and their cargo prior to transport, and thus heterologous co-expression of REEPs may be necessary to ensure proper trafficking of GPCR cargo in native cells, as originally suggested by Saito [1].

### REEPs Are Localized to ER Membranes

Prior research has demonstrated conflicting localization of REEPs within cells. When REEP1 was first identified as a possible causative agent of HSP, it was suggested that REEP1 was localized to mitochondria [4]. Others found evidence that REEP1, REEP2, and REEP5 could alter ER structure [16-18] and REEP1 and REEP2 were localized to ER and/or plasma membranes [1,3,16]. More recent work has demonstrated localization of REEP3 and REEP4 within ER membranes and as regulators of nuclear envelope architecture [34]. To clarify these apparent discrepancies, we examined REEP membrane localization by combining immunofluorescent cell staining with biochemical analyses to reach our conclusion that REEP1/2/6 are ER resident proteins, not found at the plasma membrane.

Though multiple cell types were utilized for heterologous expression, some common themes have emerged. REEP1 has been shown to co-localize with ER markers (calnexin and Sec61ß and ßtubulin, but not GM130, a Golgi marker [16]. We observed similar ER expression for REEP1/2/6, based upon co-localization studies with calreticulin, giantin (a Golgi marker) and the ER luminal dye ER Tracker™ Blue/White DPX (Figures 2 and 3). Additionally, we demonstrated that members of both REEP subclasses have similar subcellular localizations within the ER (Figure 4). REEP1/2/6 did not completely co-localize with the ER resident protein calreticulin, suggesting that REEPs may partition to ER subdomains, as seen previously with other ER marker proteins [28,29]. *In vivo* biotinylation and SGMF analyses clearly demonstrated that REEPs were not detected at the plasma membrane, only ER membranes, supporting a possible role for REEPs in intracellular trafficking. Given that ER and mitochondrial organelles form functional connections [35], we cannot state that REEPs do not interact or associate with mitochondria, only that they definitely localize to ER membranes [4,36]. Therefore, we have concluded that REEP1, REEP2, and REEP6 are ER resident proteins, based upon biochemical and immunocytochemical analyses.

**Figure 15:**
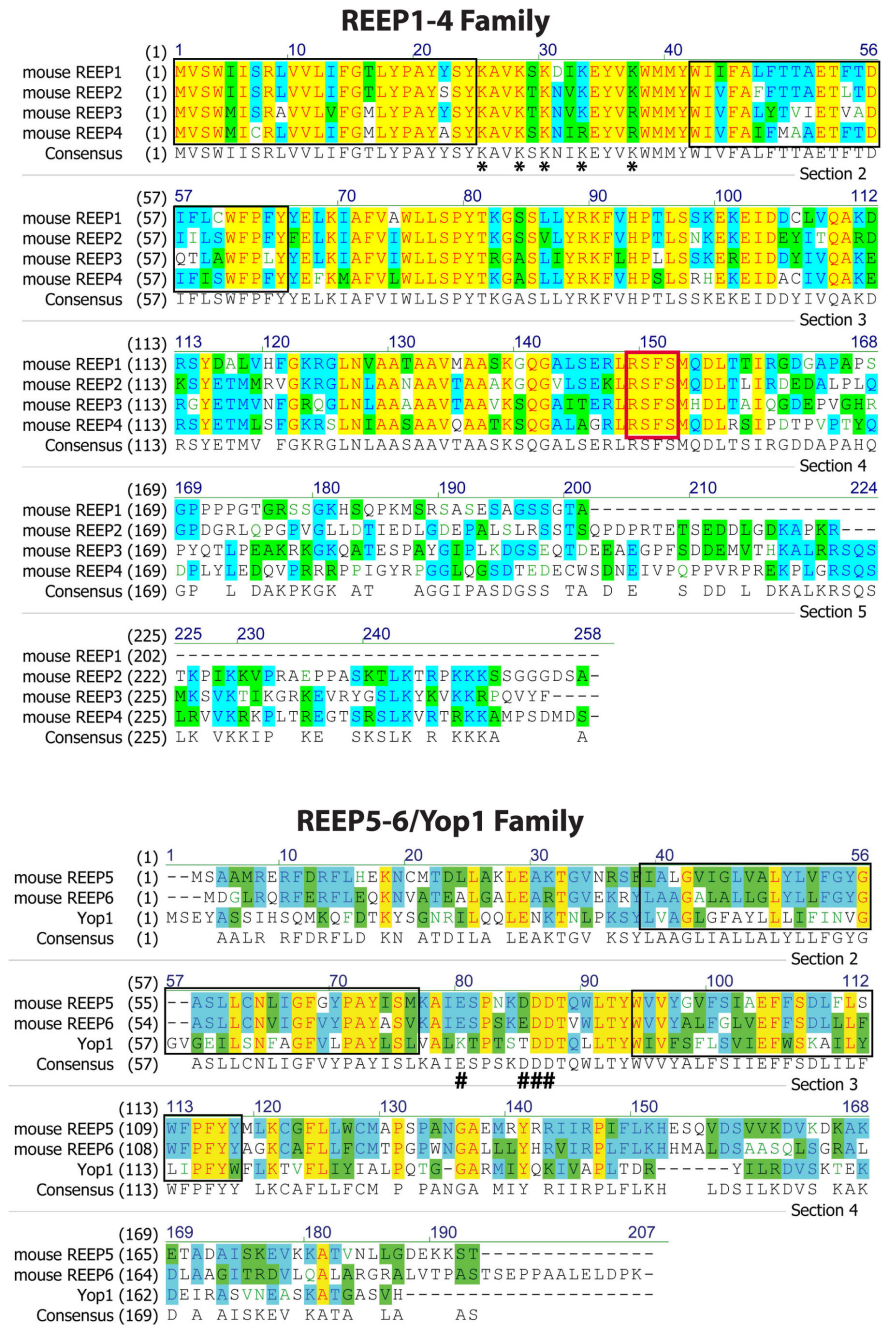
Amino acid comparison of REEP Families. The REEP family can be subdivided into two subfamilies, REEP1-4, and REEP5-6. Yeast Yop1 is most similar to the latter subfamily and has been included in the alignment. Residues completely conserved in each subfamily are highlighted in yellow, whereas partially similar residues are highlighted in blue (consensus residue derived from a block of similar residues at a given position) or green (consensus residue derived from the occurrence of greater than 50% of a single residue at a given position). Hydrophobic segments are boxed in black. The conserved 14-3-3 binding site (RSXpS) found in REEP1-4 is boxed in red. Conserved positively charged residues postulated to be involved with microtubulin binding in REEP1-4 are demarcated (*), whereas conserved negatively charged residues in REEP5-6/Yop1 are also shown (#) [34]. Alignment performed using Vector NTI v.11 (Invitrogen). GenBank protein accession numbers utilized were: mouse REEP1 (Yip2a): NP_848723 mouse REEP2 (Yip2d): NP_659114 mouse REEP3 (Yip2b): NP_848721 mouse REEP4 (Yip2c): NP_850919 mouse REEP5 (Yip2e): NP_031900 mouse REEP6 (Yip2f): NP_647453 Yop1: NP_0153

When originally described by Saito [1], carboxyl terminus Flag-tagged REEP1 was localized to the plasma membrane (with an extracellular carboxyl terminus), based upon immunofluorescent staining with anti-Flag antibody M2. However, the plasma membrane was not delineated by either biotinylation or other plasma membrane marker proteins. Using similar Flag-tagged REEP constructs, we have shown that intracellular staining of REEP1, REEP2, and REEP6 can be seen following PFA fixation under non-permeabilized and permeabilized conditions. Similarly, others examined amino and carboxyl HA-epitope tagged REEP2 localization in non-permeabilized cells [3], leading to the conclusion that REEP2 was expressed at the plasma membrane as a single transmembrane domain protein with an extracellular amino terminus. However, the plasma membrane was not delineated with a surface marker, nor were permeabilized cells studied in parallel for co-localization with possible intracellular organelles. Additionally, they showed cell surface biotinylation of REEP2, though selective biotinylation of cell surface proteins (e.g. mature glycosylated receptors) was not demonstrated. However, prior work has demonstrated that REEP1 and REEP5 having a dual hairpin structure that is inserted into the ER, with cytoplasmic amino and carboxyl termini, contrary to the above model of REEP2 as single transmembrane domain protein [16,17]. Of note, there is a high degree of homology between REEP1 and REEP2 membrane spanning domains (Figure 15).

Together, our immunofluorescent and biochemical studies reveal a consistent theme of ER localization of REEP1/2/6, as the site for α2C AR interactions. Co-expression of this subset of REEPs enhanced the expression of a minimally glycosylated form of the receptor, that interacted with the carboxyl tail of REEP1/2/6. The small amount of REEP1/2/6 co-localization with α2C ARs (Figure 5), most likely represents the site of interaction of the minimally glycosylated form, which is a minor contributor to the total pool of mature and immature glycosylated α2C ARs, possibly accounting for the low amount of co-localization seen. The lack of α2A AR co-localization with REEP1/2/6 is consistent with the lack of interaction between these two proteins, as observed by co-immunoprecipitation (Figure 13).

### REEPs are ER membrane shaping adapter proteins

ER structure is in part regulated by various integral ER membrane proteins, including REEPs, which has lead to their description as “ER morphogens” [18]. Other integral membrane proteins that also utilize a similar hairpin topology to create high membrane curvature include the reticulon (Rtn) and caveolin families [37,38]. Caveolins are plasma membrane hairpin adapter proteins that can bind multiple protein partners and generate signaling complexes for GPCR recycling and trafficking [24,39]. Thus protein families that both shape membranes and act as adapters may represent a novel paradigm in membrane organization [24]. If REEPs represent a new class of membrane shaping adapter proteins, then one would expect that they: 1) localize to a specific membrane type, 2) interact with other proteins via specific domains, and 3) show specificity in their interactions and effects on cargo proteins. By utilizing α2 ARs as a model, we have been able to demonstrate that REEP1, REEP2, and REEP6 can fulfill the requirements for membrane shaping adapter proteins, in that they 1) localize to ER membranes, 2) interact with α2C ARs via their carboxyl termini, and 3) show specificity for α2C over α2A ARs.

As membrane shaping adapter proteins, REEPs would not only affect cargo trafficking but also alter ER structure. It has been well demonstrated that expression of various REEP/Yop1 homologs can lead to enlargement and tubulization of the ER [18], which would lead to increased ER membrane surface area. Our FACS analysis clearly demonstrates that REEPs enhance ER cargo capacity, consistent with REEP-induced increase in ER tubulization [16-18]. Specific interactions with REEPs and cargo proteins (e.g. specific GPCRs) may modulate intracellular processing (e.g. glycosylation) and trafficking, thus further affecting cargo expression.

In order to function as adapter proteins, REEPs would require protein interaction domains. All members of REEP1-4 have a MTB (microtubule binding) domain and a conserved 14-3-3 binding site in the carboxyl terminus (RSXpS or RXXXpS, where pS represents phosphoserine) (Figure 15) [40]. Recently, a conserved, positively charged region between the two hairpin domains of REEP1-4 has been identified as an additional determinant of microtubule binding; REEP5-6/Yop1 have a negatively charged domain within this region [34]. An analysis of the 14-3-3 phosphoproteome and interacting proteins identified REEP4 [41,42]; phosphorylation of REEP4 at this site increased 14-3-3 binding. Interestingly, REEP5-6, which are most similar to Yop1, have neither a MTB nor 14-3-3 binding site. In general, the most divergent portions of REEPs are their carboxyl termini, thus specificity of interaction and function may be encoded in this region. Recent work has suggested that REEPs may have evolved by gene duplication to become 14-3-3 dimer binding lynchpins that integrate multiple cellular signaling systems [43]. Thus, REEP1 mutations that alter this region may cause HSP due to a loss of specific protein interactions important for neuronal function, without changing REEP1 ER insertion.

### REEP Homologs and Orthologs as Regulators of Intracellular Trafficking

Our understanding of REEP function is based in part on their similarity to other proteins, such as Yop1 and HVA22. Initially, yeast two hybrid methods were used to identify an essential yeast gene termed Ypt-interacting protein 1, or Yip1 [44]. Following the identification of Yip1, several other Ypt and Yip1-interacting proteins were identified, including Yop1 (mammalian REEP or Yip2), Yipf, and Yif [8,45,46]. All members of the Yip family share a similar membrane topology with hairpin domains and extended amino and/or carboxyl termini, though they differ in their subcellular localizations (reviewed in Reference 7).

As members of the Yip family, REEPs may have similar intracellular functions that have yet to be described. Yeast Yip1 was shown to be involved with early stage ER/COPII vesicle budding, the major ER-Golgi transport vesicle and multiple Yip1 family members have been identified in purified COPII vesicles [9,14]. Also, Yop1 can immunoprecipitate specific Ypt proteins, further suggesting a role for this family in intracellular membrane trafficking [8]. Though only recently cloned, the mammalian homologs of many Yip family members appear to have similar cellular functions as their yeast counterparts. For example, Yip1A is enriched at ER exit sites, the site of COPII vesicle biogenesis, and binds COPII proteins (Sec23 and Sec24) [47]. If the function of Yips has been maintained through evolution, then REEPs could serve a similar role in neuronal vesicle trafficking.

While screening for glutamate transporter (EAAT3) interacting proteins, Yip6a and Yip6b were identified [11,33]. Further analysis of this interaction revealed that expression of Yip6b led to accumulation of a lower molecular weight non-glycosylated form of EAAT3, which required cytoplasmic interactions of the Yip6b carboxyl terminus with EAAT3. Similar interactions between Yip6a/b were identified with other excitatory amino acid transporter family members. Our work with α2 ARs would suggest that REEPs may have similar functions in intracellular transport. We demonstrated that co-expression of various REEPs with α2C ARs can lead to enhancement of minimally or non-glycosylated forms of the receptor and that REEP1 can interact with these forms via its carboxyl terminus. These results would suggest a possible role for REEPs in ER-Golgi vesicular transport regulation, as has been seen with other Yip family members.

### REEP1 Mutations and HSP

Neurons have developed a sophisticated system to transport and deliver membrane proteins from the ER to their dendritic and axonal sites of action. Membrane trafficking defects can lead to neurodegenerative diseases, such as HSP, a progressive neurodegenerative disorder of corticospinal tract neurons [48,49]. To date, over twenty mutations in REEP1 have been linked to HSP [4,50,51]. These mutations include point mutations and frame shifts which lead to premature truncations of the carboxyl terminus, possibly affecting MTB, 14-3-3 and potential phosphorylation sites or other binding domains. The hydrophobic hairpin domains of REEPs are necessary for membrane interactions with atlastin-1, M1-spastin, and reticulons. Missense mutations that alter specific amino acids in the hydrophobic hairpin domains have been identified, however the effect of these REEP1 mutations on function and ER interactions have not been fully elucidated. Recently, it has been demonstrated that mutations in dynamin2, which cause the neurodegenerative disorder Charcot-Marie-Tooth disease, may lead to a delayed axonopathy because of a loss of specific cargo trafficking, not a generalized loss of motor protein function [52]. Thus, some REEP1 forms of HSP may cause an improper loss of ER tubulization/structure (hairpin mutations); however a loss of specialized protein-protein interactions (carboxyl terminus) may also play a role in the disease.

Careful examination of the literature combined with our work on α2 ARs strongly suggests that some REEPs are not merely ER shaping proteins or morphogens, but should be reclassified as ER membrane shaping adapter proteins, similar to reticulons and caveolins [24]. REEP1/2/6 have been shown to affect ER membrane curvature and structure, and the work presented here demonstrates that they can enhance ER cargo capacity and specifically interact with cargo proteins to modify processing and trafficking. It remains to be determined how REEPs interact with cargo (i.e. the domains and/or proteins involved), if REEPs interact with Rabs or other transport/vesicle proteins, and what role REEP phosphorylation plays in its function. Finally, it is plausible that some REEP1 mutations may lead to HSP pathogenesis due to dysfunction of these other processes.

## Material and Methods

### Materials

Nonidet P-40, Triton X-100, benzamidine, ethylenediaminetetraacetic Acid (EDTA), trishydroxymethylaminomethane (Tris), phenylmethylsulphonyl fluoride (PMSF), pepstatin, aprotinin, cycloheximide, sodium orthovanadate (Na _3_VO_4_), saponin, and paraformaldehyde (PFA) were obtained from Sigma-Aldrich Corp. Leupeptin was obtained from Roche.

### Plasmid Constructs

Wild-type mouse REEP1-5 constructs were donated by Dr. Harumi Saito of Duke University Medical Center [1]. Mouse REEP6 was cloned from mouse embryo cDNA (Ambion) by PCR using specific primers and cloned into pcDNA3.1 and verified by sequencing. REEP1, REEP2, and REEP6 were epitope tagged on the extreme carboxyl terminus with a Flag epitope (DYKDDDDKA) and REEP1 was also epitope tagged on the extreme carboxyl terminus with an HA epitope (YPYDVPDYA), using QuickChange Site-Directed mutagenesis per manufacturer’s recommendation. All α2 AR constructs possess an extracellular HA epitope tag at the extreme amino terminus. It has previously been shown that this tag does not interfere with receptor trafficking, folding or function [53]. REEP1(Arg113X) was constructed from mouse REEP1 using QuickChange Site-Directed mutagenesis.

### Cell culture and transfection methods

HEK 293A, Rat1, and NRK cells were cultured at 37°C and 5% carbon dioxide with Dulbecco’s modified Eagle’s media (GIBCO) supplemented with 10% Fetal Bovine Serum (GIBCO). Transient expression of HA-tagged α2-ARs and Flag-REEPs was achieved using Effectene (Qiagen) transfection reagent per manufacturer’s recommendation. For control “co”-transfections, empty pcDNA3.1 plasmid was used.

### Fluorescent Cell Imaging

Transfected cells (100-150,000) were seeded on sterile poly-D-lysine coated glass coverslips [25]. Forty-eight hours post-transfection, cells were rinsed three times with Phosphate Buffered Saline supplemented with Calcium and Magnesium (PBS-CM). Cells were fixed for five min with 4% PFA at room temperature (RT). Blocking agent (5% dry milk, 2% Goat Serum, 50 mM HEPES pH 7.4 in PBS-CM) was used to reduce nonspecific antibody activity. For permeabilized experiments, NP40 with a final concentration of 0.2% was added to the blocking solution. Antibody applications were performed in blocking solution for one hr at RT. Various combinations of the following antibodies were used: mouse monoclonal anti-HA (16B12) (1:500; Covance), rabbit polyclonal anti-HA (1:500; IC Labs), rabbit polyclonal anti-Flag (1:500; Cellular Signaling), rabbit polyclonal anti-calreticulin (1:1000; Abcam) or monoclonal anti-giantin (1:500; Abcam). Stained cells were washed 3x with PBS-CM at five min intervals and blocking solution was re-applied to the cells for 20 min. Secondary antibody was applied for one hr in the dark, at RT. Secondary antibodies used included goat anti-mouse Alexa 594 or goat anti-rabbit Alexa Fluor 488 (1:1000; Invitrogen). After secondary labeling, cells were rinsed 3x with PBS-CM at five min intervals in the dark and mounted using Vectashield Hard Set mounting medium H-1500 with DAPI (Vector Laboratories, Inc.). Alternatively, various fluorescent conjugated antibodies were utilized for direct labeling: FITC-conjugated 16B12 (anti-HA, 1:500; Covance), Alexa 594-conjugated M2 (anti-Flag, 1:500; Sigma), Alexa 488-conjugated 16B12 (anti-HA, 1:500; Covance), or Cy3-conjugated M2 (anti-Flag, 1:500; Sigma). With directly conjugated antibodies, cells were washed 3x with PBS-CM at five min intervals in the dark and mounted using Vectashield as described above and examined by either wide field or confocal immunofluorescent microscopy.

### *In vivo* biotinylation and immunofluorescent analysis

Forty-eight hours after transfection, cells were washed three times with ice cold PBS with calcium and magnesium. Cell surface proteins were labeled with ice cold 50 µM EZ-Link Sulfo-NHS-SS-Biotin (Pierce) in PBS pH 7.6 for 30 minutes at 4 C, per manufacturer’s instructions. Sulfo-NHS-SS-biotin labels free primary amines of proteins at the cell surface with minimal membrane permeability. After incubation, cells were washed twice with ice cold PBS and the biotinylation reaction was then quenched by addition of 50 mM glycine in PBS pH 7.5 for 15 minutes at 4 C. The cell preparation was then washed twice with PBS and fixed with 4% PFA for 10 minutes at room temperature. Subsequently, cells were washed three times with PBS at 10 minute intervals. The preparations were blocked with BSA blocking buffer contain 0.2% NP40 for 30 minutes at room temperature. Cells were then labeled for one hour with fluorescently conjugated Avidin-Alexa 594 (Invitrogen) to identify biotinylated cell surface proteins and either M2-FITC or anti HA-Alexa 488 antibody for REEP proteins or α2 ARs, respectively. The cell preparation was then washed three times with PBS at 10 intervals and mounted onto slides using Vector Shield immunomount medium and examined by confocal microscopy.

### ER Tracker™ – Blue/White DPX dye labeling of ER tubules

The ER structure was identified by labeling live cells with ER Tracker™, Blue/White DPX dye (Invitrogen). Forty-eight hours after cells were transfected on coverslips (described above), cell were washed twice with Hank’s Balanced Salt Solution (HBSS) with calcium and magnesium. Cells were incubated with 1 µM ER Tracker™ Blue/White DPX dye in HBSS for 30 minutes in the dark at 37C with 5% carbon dioxide. The cells were then washed twice with HBSS and fixed with 4% PFA for 10 min at room temperature. The preparations were washed three times with PBS at 10 minute intervals and then blocked and permeabilized with a solution containing 50 mM HEPES pH 7.4, 3% BSA, 2% goat serum, 0.1% saponin, in PBS. The cells were then labeled with M2-FITC antibody for 1 hour in blocking solution. Cells were washed three times at 5 minute intervals with PBS contain 0.1% saponin, followed by two washes with PBS, and mounted on slides using Immu-Mount (Shandon) hard mount medium. Cells were imaged with confocal microscopy as described below. ER Tracker™, Blue/White DPX dye was detected with a 405 nm Diode laser.

To quantify the extent of ER Tracker™ and REEP protein co-localization Pearson’s correlation coefficients were calculated using Volocity 6.1.1 software (PerkinElmer). For cell analysis, the region of interest (ROI) encircling the cell was performed and the background was manually determined by selecting a ROI outside the cell field. Co-localization calculations were performed by applying background correction to the thresholds. Global Pearson’s correlations and co-localization coefficients m_1_ and m_2_ were calculated, representing the extent of ER Tracker™ co-localization with REEPs and REEP co-localization with ER Tracker™ respectively.

### Wide field and confocal immunofluorescent microscopic analysis

For wide field microscopic analysis, mounted cells were observed using a fluorescence microscope (Zeiss Axioplan 2 imaging; Carl Zeiss, Inc.) with a 63x 1.4 NA oil immersion apochromatic lens (Carl Zeiss, Inc.). The cell images were acquired by digital camera (Roper Scientific RTE/CCD) using IPlabs software (Macintosh version). The image brightness and contrast were adjusted using Adobe Photoshop CS3 version 10 (PC version). The confocal cell images were obtained using a Leica SP2 AOBS Confocal microscope with 63X 1.32 NA HCX PL APO oil lens. Image acquisition was done using Leica Confocal v2.5 build 1347 software. Images were processed as described above.

### *In vivo* biotinylation and avidin pull down analysis of cell surface proteins

Cells were grown in 10 cm dish to about 60 to 70% confluency, prior to transfection with empty vector, Flag-REEP1, -REEP2, or -REEP6 alone or in combination with either HA-α2A or -α2C ARs. After forty-eight hours, cells were washed twice with ice cold PBS with calcium and magnesium and then labeled with ice cold 50 µM EZ-Link Sulfo-NHS-SS-Biotin (Pierce) in PBS pH 7.6 for 30 minutes at 4 C, per manufacturer’s instructions. The biotinylation solution was removed and cells were washed twice with ice cold PBS and the biotinylation reaction was quenched with 50 mM glycine in PBS pH7.5 at 4C for 15 minutes. Cells were washed two times with PBS and harvested with modified RIPA buffer to produce cell lysates. Protein concentration was determined using DC Protein assay kit (BioRad) with BSA as the standard. Biotinylated proteins were pulled down using Dynabeads M-280 Streptavidin (Invitrogen). Dynabeads were washed once with RIPA buffer and then incubated with 1 mg of cell lysate at 4 C overnight. After protein binding, Dynabeads were washed three times with RIPA buffer and proteins were eluted off the beads using 50 l of 2X SDS sample buffer. Protein samples were heated to 45 C for 30 minutes, run on 10% SDS-PAGE gel, and proteins in the gel were transferred to nitrocellulose using standard methods and subsequently analyzed by immunoblotting.

### Sucrose Gradient Membrane Fractionation (SGMF)

HEK293A cells were transfected with various combinations of Flag-REEP and HA-α2 AR cDNA constructs. Forty-eight hours after transfection, HEK293A cells were washed two times with PBS with 1 mM EDTA, and harvested in 5 ml of PBS with 1 mM EDTA and centrifugation at 500 x g for 5 min. Cell pellets were resuspended in Fractionation Homogenization Buffer (FHB: 250 mM Sucrose, 20 mM HEPES pH 7.4, 2 mM EDTA pH 7.4, 2 µg/ml Aprotinin, 1 mM Benzamidine, 5 µg/ml Leupeptin, 1 µg/ml Pepstatin, 1 mM PMSF, 0.5 mM Na _3_VO_4_ and 0.03 mM Cycloheximide in water) and homogenized with 30 strokes of a 1 ml Dounce Style Tissue Grinder (Wheaton). Homogenate was centrifuged at 500 x g for 5 min to clear debris. Cleared homogenate was brought to 1.4 M sucrose via addition of 2 M sucrose buffer (2M sucrose, 20 mM HEPES pH 7.4, 2 mM EDTA pH 7.4 in water). Samples were loaded onto a discontinuous sucrose gradient (2M, 1.6M, 1.4M (Sample), 1.2M, 1.0M, 0.8M, 0.6M). Gradients were centrifuged at 4°C, 37000 rpm for 1.5 hrs in a Beckman L8-80M ultracentrifuge (SW41Ti rotor) and collected into eleven fractions. Fractions were solubilized by addition of 10% triton X100 to final concentration of 1%. Next, each fraction was mixed with 5x SDS sample buffer and heated at 45°C for 30 min. 10µL of pre-gradient homogenate along with 125µL of each prepared fraction were run on 12% SDS-PAGE gels and transferred to nitrocellulose for Western Blot analysis.

### Cellular Processing of α2-AR via Deglycosylation Analysis

Eighteen hours after transient transfection of HEK293A cells, membrane protein preparations were obtained by the same procedure described in **SGMF** section except that hypotonic lysis buffer (HLB: 10 mM HEPES pH 7.4, 1mM EDTA pH 7.4) was used for homogenation, and cleared homogenate was centrifuged at 16000 x g for 45 min to obtain cell membranes. Membrane pellets were resuspended in HLB and protein concentrations were determined using a DC Protein Assay Kit (Bio-Rad) with BSA as a standard. Membrane preparations (50 to 85 µg) were treated for 4 hrs with endoglycosidase H (Endo H) or peptide-N-glycosidase (PNGase F) (New England Biolabs) per manufacturer’s recommendations. Preparations were run on 10% SDS-PAGE gels and transferred to nitrocellulose for immunoblot analysis.

### Radioligand Binding Assay

Total ligand binding was determined by saturation binding with the α2-adrenergic receptor antagonist [^3^H]RX821002 (GE Healthcare). Cell membrane pellets were obtained as described above for deglycosylation analysis section, but resuspended in binding buffer (75 mM Tris, 12.5 mM MgCl_2_, 1 mM EDTA pH 7.4). Yohimbine was used to obtain non-specific binding, as described previously [25]. Samples were transferred to Whatman GF/C glass filter paper by vacuum filtration using a Brandel M-48 Harvester. Filter paper was put in scintillation tubes and mixed with 5 ml of scintillation fluid overnight. Counts were obtained using a Beckman LS6000IC liquid scintillation counter.

### Flow Cytometry via Fluorescence Activated Cell Sorting (FACS)

HEK293A cells were grown to 70-80% confluency for transfection in 10 cm dishes. Cells were transfected using Effectene per manufacturer’s recommendation. At forty-eight hours post transfection, the cells were washed twice with 10 ml of PBS supplemented with 2 mM EDTA pH 7.4. The cells were detached from the dish with 10 ml of HBSS supplemented with 5 mM EDTA. Cells clumps were broken up by trituration using a P200 pipette tip. The cell suspension was centrifuged at 150 x g for 5 minutes at 4C. The supernatant was discarded and cell pellet was resuspended in 10 ml of PBS without EDTA. The cells were centrifuged at 150 x g for 5 minutes at 4C. The supernatant was discarded and the cells were resuspended 1 ml of PBS and fluorescently labeled using Live/Dead Fixable Violet™ fixable stain reagent (Invitrogen) per manufacturer’s recommendation for 30 minutes on ice in the dark. The cell preparations were washed twice by resuspending in 10 ml of PBS and centrifuged at 150g and 4C for 5 minutes. The supernatant was removed and the cell pellet was resuspended in 1 ml of PBS. For fixation of the cell preparations, 1 ml of 4% paraformaldehyde was mixed into the cell suspension and incubated for 10 minutes at RT in the dark. The fixed cell samples were divided into two 1 ml aliquots and placed into two separate eppendorf tube (non-permeabilized and permeabilized). The samples were centrifuged at 600 x g for 5 minutes in the dark at RT. The samples were subsequently washed twice by resuspending cell pellets in 1 ml of PBS and centrifuging at 600 x g for 5 minutes in the dark and removing the supernatant. For the non-permeabilized condition, cell preparations were resuspended in 250 µl blocking buffer (PBS with 2% FBS) for 45 minutes. For the permeabilized condition, cell preparations were resuspended in 250 µl of detergent blocking buffer (PBS with 2% FBS and 0.1% Triton) for 15 minutes. The permeabilized cell preparations were then centrifuged at 2400 x g for 5 minutes at RT. The supernatant was removed from the permeabilized samples and resuspended in with 250 µl blocking buffer for 30 minutes at RT. Permeabilized and non-permeabilized preparations were centrifuged at 2400 x g for 5 minutes at RT and the supernatant was removed. For fluorescently labeling the cell expressing HA epitope tagged α2 ARs and the flag epitope tagged REEP1, 2, and 6 proteins, both the non-permeabilized and permeabilized cell preparations were resuspended in 200 µl of antibody labeling media containing: PBS-CM, 2% FBS and 16B12 Alexa-488 antibody (anti-HA, 1:500; Covance) and M2-Cy3 antibody (anti-Flag, 1:500; Sigma), respectively. Cell preparations were labeled in the dark for 30 minutes. The cell preparations were washed thrice by centrifuging at 2400 x g, removing the supernatant, and resuspending with 1 ml of PBS with 2% FBS. Cell preparations were resuspended in 1 ml of PBS prior to analyzing on LSR-II FACS. For setting the appropriate size and fluorescent channel gates and compensation matrix, cells were prepared as follows: untransfected unlabeled, untransfected live/dead violet labeled, and single transfected and labeled α2 AR (16B12-Alexa-488) and REEP (M2-Cy3) samples prepared as non-permeabilized and permeabilized preparations. Analysis of the FACS derived data, were performed using the software FlowJo (Version 7.6.34, Tree Star, Inc.). Calculation of % surface/plasma and intracellular membrane expression has been described previously [23].

### Co-Immunoprecipitation (Co-IP)

Transfected HEK293A cells were harvested using modified RIPA buffer (0.15 M NaCl, 1% NP40, 0.25% DOC, 50mM HEPES pH 7.4, 1mM EDTA pH 7.4, 2 µg/ml Aprotinin, 1mM Benzamidine, 5µg/ml Leupeptin, 1 µg/ml Pepstatin, 1 mM PMSF). Protein concentrations were determined using a DC Protein Assay Kit (Bio-Rad) with BSA as a standard. Co-IP was performed using Dynabeads Protein G (Invitrogen). Manufacturer recommendations were followed with these changes: 1 µg of either mouse M2 (Sigma) or mouse 16B12-HA antibody (Covance) was bound to beads at room temperature for ≥1.5 hours prior to addition of sample; RIPA buffer was used in place of Ab Binding & Washing Buffer; 1 mg of protein in 800 µL volume was added to the Dynabeads-Antibody complex and incubated overnight at 4°C. After binding, proteins were eluted off the beads using 50 µL of 2x SDS sample buffer, heated at 45°C for 30 minutes, and run on a 10% SDS-PAGE gel. Gels were transferred to nitrocellulose via standard protocol for subsequent analysis via immunoblot.

### Immunoblot analysis

Nitrocellulose blots were blocked with blocking solution (5% BSA, 2% Goat Serum, 50 mM HEPES pH 7.4, 0.1% Azide) for 1 hr at RT. Primary antibody was applied in blocking buffer with 0.1% tween-20 for 1 hr. Blots were rinsed 5x at five min intervals with Tris-Buffered Saline with 0.1% tween-20 (TBS-tween). Secondary infrared antibody was applied in blocking buffer with 0.1% tween-20 for one hr (1:10,000). Blots were rinsed again 5x at five min intervals with TBS-tween and stored in PBS with 0.1% Azide. Images of labeled blots were obtained with an Odyssey Infrared Imaging System (LI-COR Biosciences). Color, brightness and contrast of blot images were adjusted using Odyssey software version 3.0. Various antibodies were utilized including mouse monoclonal HA antibody 16B12 (1:1000; Covance) or rabbit polyclonal HA antibody (1:1000; ICLAbs), rabbit polyclonal antibody C10 or C4 (1:300; α2A and α2C ARs respectively). Mouse monoclonal antibody M2 (1:10,000; Sigma) was used to detect flag-REEPs. Rabbit polyclonal antibodies anti-Calreticulin (1:1000; Abcam) and anti- Na^+^/K^+^ ATPase (1:1000; Abcam) were used to respectively label the ER compartment and plasma membrane fractions of SGMF experiments. IRDye 800CW Goat anti-Mouse IgG antibody and IRDye 680 Goat anti-Rabbit IgG antibody (1:10,000; LI-COR Biosciences) were used as secondary antibodies.

## Supporting Information

Figure S1 **Plasma membrane confocal co-localization of α2A ARs and REEPs.** HEK293A cells were co-transfected with HA-α2A AR and either empty vector (pcDNA3.1), Flag-REEP1, -REEP2, or –REEP6 cDNAs. Cells were fixed with 4% PFA, permeabilized, and examined by confocal microscopy forty-eight hrs post-transfection. α2A ARs were stained with anti-HA mAb (16B12) and Alexa 594 conjugated-anti mouse secondary antisera; REEPs were stained with rabbit anti-Flag polyclonal antisera and Alexa 488 conjugated anti-rabbit secondary antisera. **Left**: To enhance detection of possible α2A AR/REEP co-localization, confocal images were focused on plasma membrane planes, the predominant site of α2A AR expression. Note predominant plasma expression of α2A ARs. **Middle**: Immunolabeling of REEPs identified a slight intracellular reticular/punctate pattern. Note reduced expression of REEPs in plasma membrane planes (compared to ER planes, Figure 5). **Right**: REEPs did not overlap with plasma membrane localized α2A ARs. Absence of α2A AR (vector control) did not alter REEP localization. Representative of three separate transfections. Scale bars: 25 µm.
(TIF)
