# Correction: REEPs Are Membrane Shaping Adapter Proteins That Modulate Specific G Protein-Coupled Receptor Trafficking by Affecting ER Cargo Capacity

**DOI:** 10.1371/annotation/6f86410c-63c3-4fcd-b1cb-9fd8d2ea95d0

**Published:** 2013-12-17

**Authors:** Susann Björk, Carl M. Hurt, Vincent K. Ho, Timothy Angelotti

This article was republished on December 16, 2013 because of illegible figures. Please download this article again to view the figures. 

