# Supplementary figures and images for: REEPs Are Membrane Shaping Adapter Proteins That Modulate Specific G Protein-Coupled Receptor Trafficking by Affecting ER Cargo Capacity
Source: PLoS One. 2013 Oct 2;8(10):e76366. doi: 10.1371/journal.pone.0076366 (PMC3788743; doi:10.1371/journal.pone.0076366)

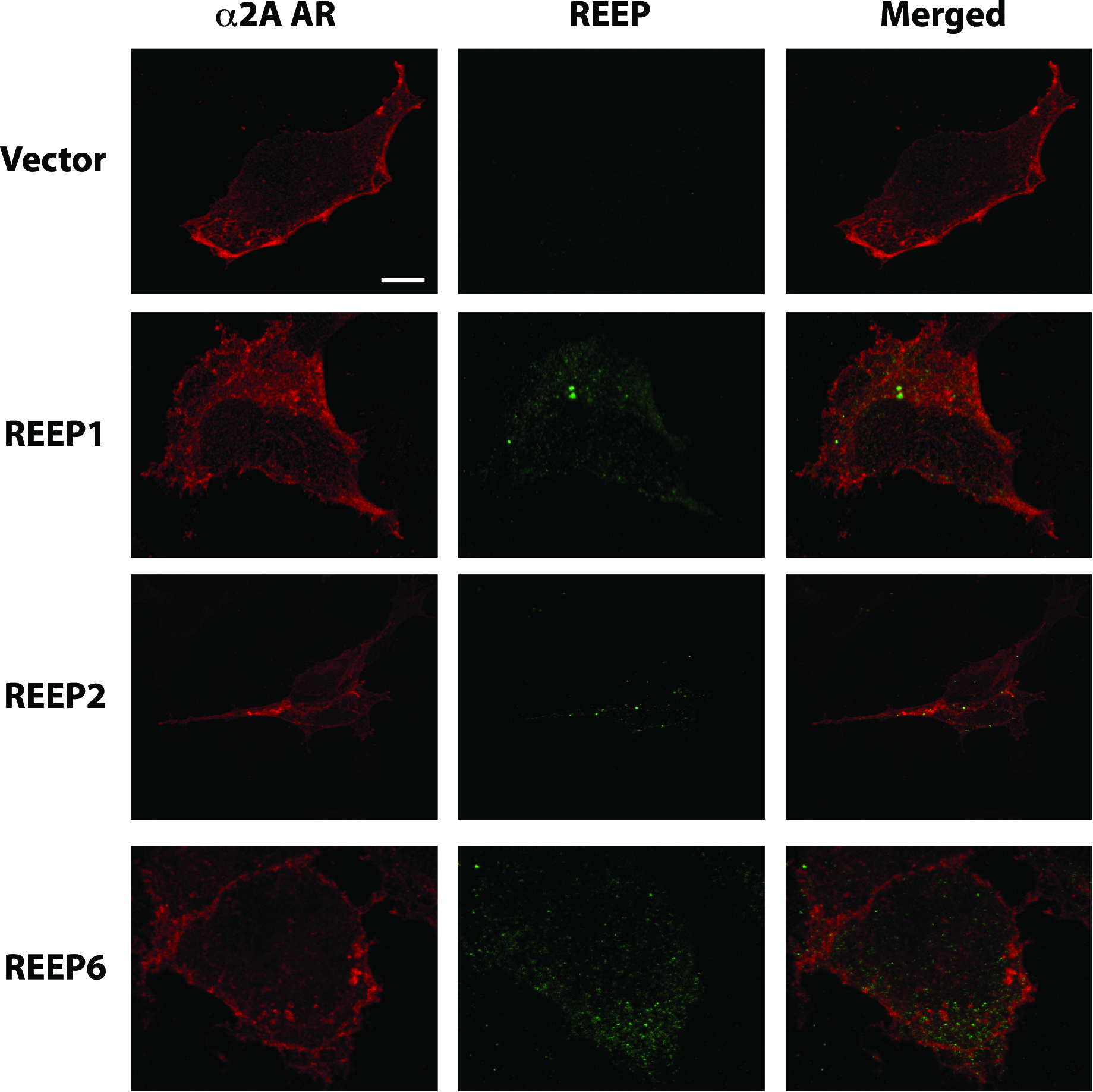

Supplement: Figure S1 — Plasma membrane confocal co-localization of α2A ARs and REEPs. HEK293A cells were co-transfected with HA-α2A AR and either empty vector (pcDNA3.1), Flag-REEP1, -REEP2, or –REEP6 cDNAs. Cells were fixed with 4% PFA, permeabilized, and examined by confocal microscopy forty-eight hrs post-transfection. α2A ARs were stained with anti-HA mAb (16B12) and Alexa 594 conjugated-anti mouse secondary antisera; REEPs were stained with rabbit anti-Flag polyclonal antisera and Alexa 488 conjugated anti-rabbit secondary antisera. Left: To enhance detection of possible α2A AR/REEP co-localization, confocal images were focused on plasma membrane planes, the predominant site of α2A AR expression. Note predominant plasma expression of α2A ARs. Middle: Immunolabeling of REEPs identified a slight intracellular reticular/punctate pattern. Note reduced expression of REEPs in plasma membrane planes (compared to ER planes, Figure 5). Right: REEPs did not overlap with plasma membrane localized α2A ARs. Absence of α2A AR (vector control) did not alter REEP localization. Representative of three separate transfections. Scale bars: 25 µm. (TIF) [file pone.0076366.s001.tif]
